# Computing with DFT Band Offsets at Semiconductor Interfaces: A Comparison of Two Methods

**DOI:** 10.3390/nano11061581

**Published:** 2021-06-16

**Authors:** José C. Conesa

**Affiliations:** Instituto de Catálisis y Petroleoquímica, CSIC, 28049 Madrid, Spain; jcconesa@icp.csic.es

**Keywords:** band alignment, hybrid functional, vacuum, epitaxy

## Abstract

Two DFT-based methods using hybrid functionals and plane-averaged profiles of the Hartree potential (individual slabs versus vacuum and alternating slabs of both materials), which are frequently used to predict or estimate the offset between bands at interfaces between two semiconductors, are analyzed in the present work. These methods are compared using several very different semiconductor pairs, and the conclusions about the advantages of each method are discussed. Overall, the alternating slabs method is recommended in those cases where epitaxial mismatch does not represent a significant problem.

## 1. Introduction

The determination of the band alignment (also called band offset) at semiconductor interfaces is a subject of high interest as it allows foresight in a number of different fields such as photovoltaics, (opto)electronics, thermoelectricity, photoelectrochemistry and photocatalysis, plasmon resonance effects, etc.; it governs indeed the direction of electron (or hole) transfer at those interfaces. This alignment can be obtained in several cases experimentally; in general this is performed using photoelectron spectroscopy (e.g., ref. [1], or the more recent ref. [2] for its application to the case of perovskite oxides), but Kelvin probe/surface photovoltage [3,4], scanning probe microscopy [5] or electrochemical impedance analysis [6] were used also to achieve this aim.

Theoretical calculations were also used for this purpose. Although in the past relatively crude methods such as effective dipole models [7], tight-binding schemes [8] or empirical rules (even recently [9]) were used, most of the approaches in the last 30–40 years use Density Functional Theory in one or another variant. One possibility to achieve this aim is to determine, for an alternating slab model of both materials, the DOS in regions far from the interface so that, by comparison with the results of bulk calculations, the position of the valence and conduction bands of the two semiconductors can be inferred [10]. In other cases, equilibration of Fermi levels was proposed [11], but this scheme might not be adequate, since the position of that level inside the bandgap is uncertain in non-doped semiconductors. Other methods using clusters [12] or nanoparticles [13] are less frequent.

Of particular interest, since they are the most used ones, are those methods that compute the electrostatic (Hartree) potential inside a slab model of the interface, and relate it to the positions of the valence and conduction bands (VB and CB in the following) computed previously for the bulk solid [14]. This approach is reasonable, even when the functionals used for bulk and slab calculations are different, as it was found generally that the difference in the distribution of electronic density obtained with different functionals for the same system, which determines the Hartree potential, is small, and the band alignments obtained in this way practically do not depend on the function with which the Hartree potential is computed (e.g., refs. [15,16]). In most cases the electrostatic potential is averaged in planes parallel to the interface, and the position of both VB and CB are located relative to the resulting 1D profile; alternatively, the Hartree potential can also be volume-averaged in the central region of each slab (e.g., Figure 4 in ref. [17]).

However, even within the methods using the Hartree potential (plane- or volume-averaged), two main approaches are followed. In one of them (e.g., ref. [18]) each semiconductor slab is facing the vacuum, obviously keeping the lateral dimensions of each 2D slab equal to those determined for the corresponding bulk phase but otherwise relaxing all the atom positions. In the other one (e.g., ref. [19]) both slabs are handled jointly in a periodic arrangement of alternating slabs without any empty region; the lateral dimensions are forced initially to coincide to some average value, and then the full structure is relaxed in both cell dimensions and atomic coordinates, except when one wants to specifically model a thin layer deposited on a massive solid, in which case the lateral dimensions are those of the latter material [20]. There are also approaches where the interface between both materials is taken into account in a mixed slab, but in addition an empty space is included [21,22,23]; in these cases, however, there may be a significant electric dipole normal to the interface in the whole cell, which may produce an electrostatic potential gradient (as can be seen, e.g., in Figure 2 of ref. [21]) making it more difficult to achieve a precise band alignment. Additionally, one should not fall into inadequacies such as those presented in ref. [23], in which according to its Figure 2 the BiNbO_4_ phase seems to be represented by a slab with the wrong stoichiometry (Bi_3_Nb_2_O_8_).

In the first of the cases mentioned in the paragraph above, the bandgaps can be assumed to be those of the individual phases, but no transfer of electronic density between them can take place; furthermore, the solid-vacuum interfaces, with their specific relaxations and spread of electronic density towards the vacuum side, do not correspond to the situation that one actually wants to model. In the second case these latter criticisms do not apply, but there is the risk that epitaxial forces distort the materials, modifying the bandgap and the VB and CB positions relative to the Hartree potential.

It is very infrequent that both situations are considered in one same work; one case known to this author is that presented in ref. [20]. This is actually the objective of the present article: to compare, for a series of very different semiconductor pairs, the two approaches, i.e., that in which both semiconductors separately face the vacuum and that using alternating slabs of them. Note that the situation, discussed relatively frequently in the literature at the DFT level, of alternating 2D layers of materials that normally are bonded only by dispersion interactions (van der Waals forces) is not discussed here, since these systems are unlikely to be prepared experimentally, especially in bulk form.

## 2. Methods

### 2.1. Software Used

In all this work the periodic DFT program VASP [24] will be used, the core electrons being modelled with the PAW method [25,26]; the Brillouin zone sampling, using a Γ-centred Monkhorst–Pack grid (with only one point in the c direction in the case of the slab models), was always chosen so as to get enough converged band offsets. In some cases, as may be needed, spin-orbit coupling, or spin-polarized calculations were used. Bader analyses, carried out for all mixed slab models on the basis of the relaxed PBE calculations, were performed using the software of Henkelman’s group at the University of Texas at Austin (TX, USA) [27]. Structures were drawn using Materials Studio 2019 software [28].

### 2.2. Cautions to Be Considered

In this exercise one has to take into account several aspects. Firstly, all surfaces should be nonpolar, i.e., they should be considered as formed by the stacking of elemental slabs with the same stoichiometry as the bulk solid and possessing no dipole moment normal to that slab (Tasker’s criterion) [29].

Secondly, both interfaces in all periodic slab models should be equivalent by symmetry; otherwise, the whole periodic cell may have an overall net electric dipole moment perpendicular to the interface, leading to distortions in the Hartree potential profile.

Thirdly, one should try to reproduce as accurately as possible the bandgaps of each semiconductor. It is well known that standard DFT at the PBE level [30] always underestimates bandgaps. Hybrid functionals such as PBE0 [31] or HSE06 [32] normally make a better job in this respect, but even in these cases they may not accurately approach the real bandgaps enough. These two functionals include a fixed fraction of Fock exchange (α = 0.25); but it was shown recently, on the basis of GW theory [33,34] that the optimal degree of mixing, in the case of the PBE0 functional at least, should be in first approximation equal to the inverse of the optical dielectric constant (α = 1/ε_∞_), i.e., should be adjusted depending on the screening properties of each material [35]. The present author recently proposed a self-consistent scheme to carry out this adjustment [36] which was successful for zinc titanates; it was subsequently verified to also hold for very different semiconductors [37,38]. However, this requires fitting self-consistently ε_∞_, which may be a tedious process because determining ε_∞_ may require rather lengthy calculations. Since what one needs is to reproduce the bandgap well, it may suffice, as carried out by several authors, to adjust α in order to well enough reproduce the bandgap at ambient temperature, and then proceed with the rest of the method. Here, this will be performed with the HSE06 hybrid functional.

Finally, one should try to study semiconductor pairs for which the nonpolar surfaces can be adjusted in the alternating slab model with minimal epitaxial strain, otherwise one could distort the structures modifying the bandgaps so that there would no longer be a certainty that the band alignment is correct. A problem of this kind appeared in ref. [39], in which one same semiconductor, anatase TiO_2_, gave bandgaps differing by 0.4 eV, depending on the surface orientation used for the mixed slab.

### 2.3. Development of the Models

The primitive cells of all bulk semiconductors were first relaxed at the PBE level in both cell dimensions and atomic coordinates to get the energy minimum. Linear combinations of lattice vectors were determined, if necessary, to ensure that the ab plane coincided with the interface plane chosen in each case. Then hybrid functionals of the HSE06 type were used, including an amount α of Fock exchange appropriate to get the experimental bandgap as measured at ambient temperature; in doing these HSE06 calculations, cell dimensions and atomic coordinates were kept constant. The 1D profile of the Hartree potential (always multiplied by the electron charge) averaged in planes parallel to the ab plane was then obtained, and the positions of the VB and CB relative to that potential provided by VASP in the HSE06 calculations were observed, so that they could be translated later to the plane-averaged profiles found for the slab models. Here, it is convenient to compare the VB and CB positions with the Hartree potential computed between the atomic planes, to minimize errors due to the finite grids used.

The calculations and relaxations of all the slab models were always performed with the PBE functional. In the slabs versus vacuum models the cell dimensions in the ab plane were kept fixed at the bulk values, the number of layers being chosen to ensure convergence of the Hartree potential in the innermost regions, and the c-axis dimension was fixed to values ensuring an empty space separation between slabs of 10 Å or more; otherwise, the atomic coordinates were allowed to relax fully. For the mixed slab models an averaged value of the ab plane dimensions of each phase was determined initially, and the same number of atomic layers as in the previous case was used. In addition, the relative displacement between both phases was always chosen so that the maximum number of cation-anion bonds could be formed. Then both cell dimensions and atomic coordinates were allowed to relax to the energy minimum.

In both kinds of model, the Hartree potential was plane-averaged in the ab plane (parallel to the interface), and in the alternating slabs models also the electronic density was computed and averaged in the ab plane. Calculations of the individual slabs with the same cell dimensions and atomic coordinates as in the mixed slabs were carried out as well at the PBE level, so that differences in the Hartree potential and electron densities may be obtained. The positions of the VB and CB in respect to the Hartree potential were then transferred from the bulk calculations to both slab models, thus allowing the comparison of the band offsets obtained by both methods.

## 3. Results and Discussion

### 3.1. TiO_2_ (Anatase)-ZnO Interface

Anatase TiO_2_ and hexagonal ZnO have at ambient temperature similar bandgaps of 3.2 eV (indirect) and 3.3 eV (direct at Γ point), respectively. For ZnO the (110) surface is nonpolar, and has dimensions of 5.628 and 5.207 Å; anatase TiO_2_ also has (001) as nonpolar surface (even though it is not the most stable one); and its $\sqrt{2}\times \sqrt{2}$ supercell has parameters a = b = 5.342 Å. Thus the epitaxial misfits are of 5% or lower, which justifies considering this interface.

**Figure 1:**
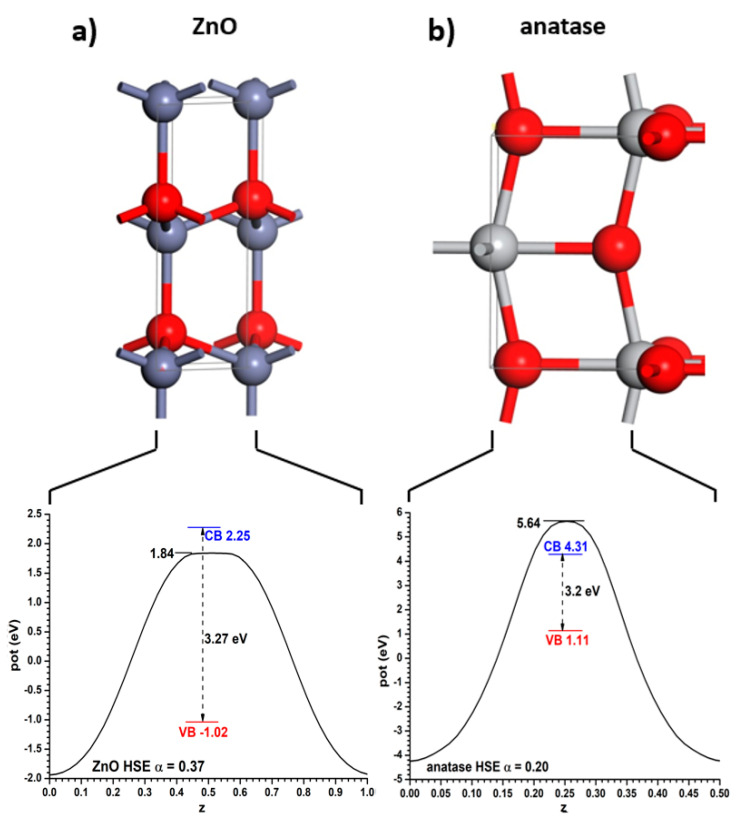
(**a**) Structure of fully relaxed ZnO, with (110) plane vertical and perpendicular to the drawing plane, displaying below it the curve of the plane-averaged Hartree potential together with the positions of the VB and CB. (**b**) the same for the anatase TiO_2_ structure, with the (001) plane located in the same way. The band positions were obtained using the HSE06 functional with the indicated α values of Fock exchange fraction. z coordinate means, here and always, the ratio between atomic positions and cell dimension.

These bulk structures were relaxed with the PBE functional, keeping in the valence space 6, 10 and 12 electrons for O, Ti and Zn, respectively. This resulted in conventional cell sizes of a = b = 3.808 Å and c = 9.703 Å (for anatase) and a = b = 3.288 Å and c = 5.305 Å (for ZnO; β = 120°). These cell lengths are a bit larger than the experimental ones, as is typical of the PBE functional. With these values, the experimental bandgaps of both semiconductors can be reproduced using the hybrid HSE06 functional if the Fock exchange mixing α is α = 0.20 (for anatase) and α = 0.37 (for ZnO). Note that if one had to use the same α value in both cases it would be impossible to obtain bandgap values equal to the experimental ones. The Hartree potential profiles averaged in planes parallel to the said interfaces, as well as the positions of the VB and CB relative to them, are given for both materials in Figure 1. Throughout this article the VB will always be represented in a red colour, the CB in a blue colour, and the relevant positions of the Hartree potential profile in a black colour. Note that the minima appear at the position of the nuclei; therefore it has been chosen to use as reference for the VB and CB the maximum position, corresponding to the planes between the nuclei. If the integration had been carried out near the nuclei it might have been subjected to important integration grid-related inaccuracies. This criterion will be followed in the rest of this article.

**Figure 2:**
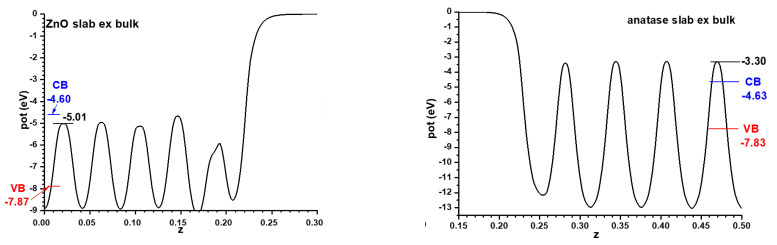
The Hartree potential profile for slabs, facing the vacuum, of ZnO (11 cation planes) and anatase (9 cation planes), indicating for this model the positions in each case of the VB and CB as transferred from the results of Figure 1. These Hartree potential profiles are displayed for only one half of the unit cells.

For the models implying semiconductor slabs facing the vacuum, symmetric slabs involving 9 cations layers and 11 cation layers were chosen for anatase and ZnO, respectively. For determining the number of layers to use, the best criterion is that in the central region of all the slabs the potential profiles are converged enough, at least in the planes between the atomic layers; this applies also to the alternating slabs method, and the criterion will be kept throughout this article. The corresponding Hartree potential profiles, after relaxing atom coordinates with fixed cell size, are given in Figure 2 together with the positions of the VB and CB transferred from the hybrid functional calculations displayed in Figure 1. In all cases only half of the unit cell is represented, since both interfaces are equivalent by symmetry. This allows the band alignment resulting from the slabs-versus-vacuum model to be inferred.

**Figure 3:**
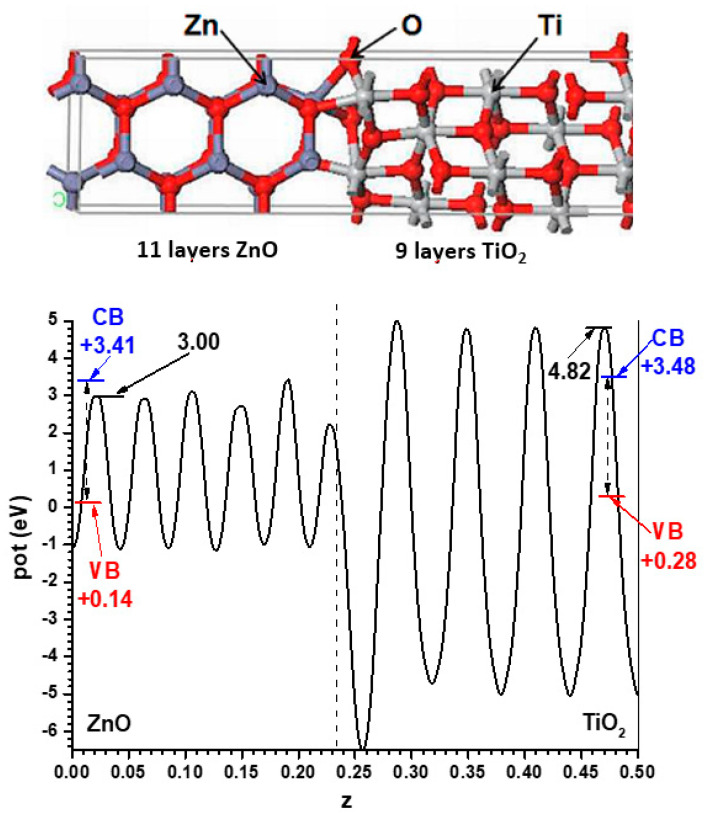
Structure of the fully relaxed mixed ZnO|anatase TiO_2_ slab (only half of the unit cell is displayed) together with the Hartree potential profile and the positions of the VB and CB transferred from the results of Figure 1.

For the model with the alternating slabs, the structure obtained after full relaxation yielded lateral dimensions of 5.496 Å × 5.361 Å; intermediate, as expected, between those of the individual phases. This structure, again showing only half of the symmetric cell, is presented in Figure 3, together with the resulting Hartree potential profile and the positions of the VB and CB transferred from the hybrid functional calculations carried out for the bulk systems, so that the band alignment predicted by this other model can be evaluated.

**Figure 4:**
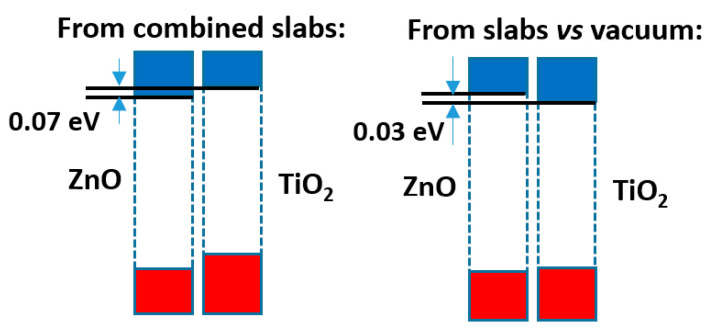
Band offsets found for the interface between ZnO and anatase TiO_2_.

The comparison of both models shows that, although the difference is not large (just 0.1 eV), the prediction of the sign of the CB offset, shown in Figure 4, is opposite: in the slabs/vacuum case the CB of ZnO is predicted to lie a bit higher than that of anatase, while in the alternating slab model the reverse happens. The outcome of the latter case agrees with the result that the present author reported in an earlier work [40], also using the alternating slabs model (even though in that case a different software was used: the CRYSTAL program, based on atom-centred basis sets), which explained the then recent result [41] that a ZnO-based dye sensitized photovoltaic cell improved its efficiency if a very thin layer of anatase TiO_2_ was deposited on the ZnO nanoparticles, which was interpreted there assuming that anatase formed with its CB a barrier preventing the recombination of the excited electrons with the hole remaining in the dye. Now it is seen that the model using slabs against vacuum would have given different results.

**Figure 5:**
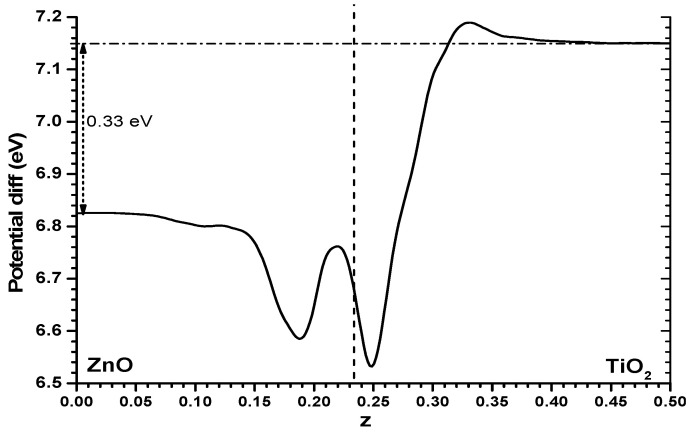
Difference between the Hartree potential profile of the alternating slabs model (for the ZnO|anatase TiO_2_ interface) and the sum of the two individual slabs facing vacuum (with cell dimensions and atomic coordinates kept fixed). As in previous cases only one-half of the cell is shown.

It is worthwhile trying to understand this difference between both models. One way could be to examine the Hartree potential profile for the alternating slabs model and obtain the difference between it and the sum of the Hartree profiles of the individual slabs, while keeping the same cell size and atomic positions. The difference, shown in Figure 5, is not large, it is certainly much smaller than the values of the full profile shown in Figure 3; but one finds that the Hartree potential imbalance between one phase and the other shown in Figure 5 (0.33 eV) is clearly higher than the difference in results between both models (just 0.1 eV). Some other effects must be at work. Very probably the reason is that while in the slabs versus vacuum model there will be a spill of electronic density towards the vacuum side which may reach different distances in the ZnO and TiO_2_ cases (and note that this will appear as well in the alternating slabs model, with fixed atomic positions involved in the said subtraction of Hartree potential profiles), this is not possible in the alternating slabs case, where in addition the Pauli exclusion principle will force a rearrangement of the individual wavefunctions. The mentioned Hartree potential difference is thus not useful to understand the difference between both methods of estimating the band offset. In the following, this difference of Hartree potential will not be considered further.

**Figure 6:**
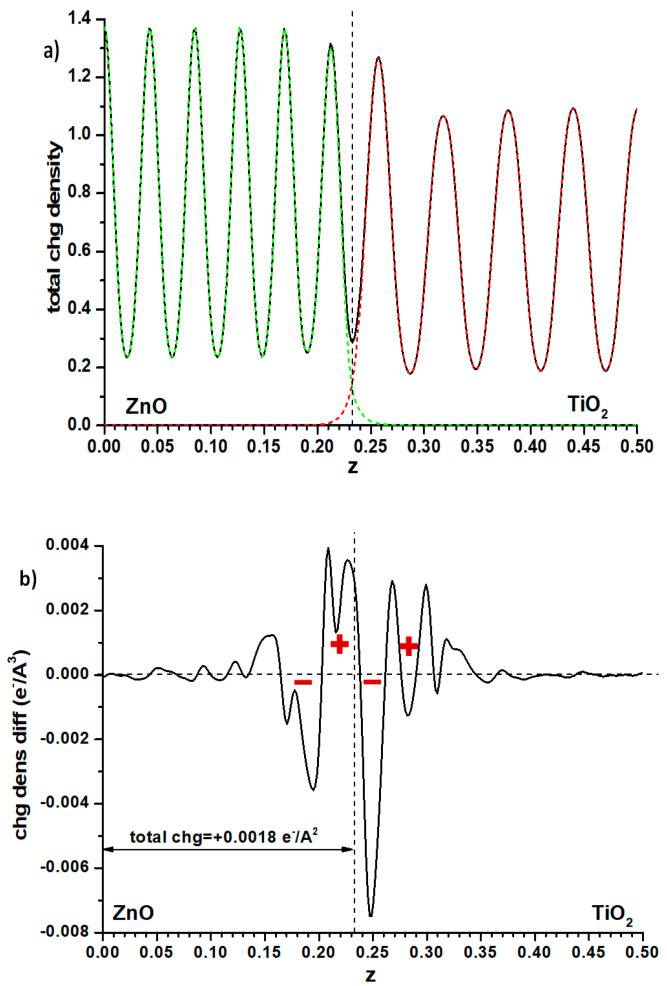
(**a**) Electron density distribution in the ZnO|anatase TiO_2_ interface, averaged in planes parallel to the latter; the same distribution for the individual slabs contacting the vacuum, with the cell dimensions and atomic coordinates kept fixed, is included as well. (**b**) Difference between the full electronic density profile and the sum of the two individual contributions given in (**a**). The vertical dashed line indicates, in all cases, the minimum of electronic density at the interface.

Another possibility is to examine the plane-averaged profile of electronic density and subtract from it the sum of the individual slabs that remain unchanged, as for the Hartree profile, the cell size and atomic positions. This may reveal some flow of electron density between both phases, perhaps explaining the effect. Again, as seen in Figure 6 where the full electronic density given by the PAW method is compared with the said difference, the effect is small. The integration of this difference, measured (somewhat arbitrarily) up to the point of the minimum in total electron density at the interface position (marked with a dashed line in that figure), implies that a small charge (0.0018 e^−^/Å^2^) has flown from anatase to ZnO. This should raise the electron levels in ZnO, contrarily to what one could think by looking at the difference between both methods of calculation of the band offsets (Figure 4). However, examining the electron density difference curve more closely, one observes that the electron density distribution implies the presence, at both sides of the interface, of a significant dipole (marked with red signs in Figure 6b) accumulating electronic density more towards the anatase side; it may be this dipole, rather than the net amount of charge transferred, which causes the electron levels of the anatase side to rise.

Finally, one can examine the Bader charges at the atoms in one and another side of the alternating slab model of the interface. Here of course the situation of slabs versus vacuum is not taken into account. One then finds that a net electronic charge of 0.006 e^−^/Å^2^ is transferred to the anatase side. The difference with the result found when considering the electronic density difference (Figure 6) may be due to different factors. The Bader charges use basins of electronic density the frontiers of which may not coincide with the interface plane. Furthermore, Bader charges are just single values that do not take into account the possibility that the Bader basins may include an uneven distribution of electronic density, i.e., may have dipoles which influence the Hartree potential profiles; for this reason, in the rest of the cases presented here, the profile of electronic density differences will still be shown, to verify whether they may present the dipole effect shown in Figure 6.

In any case, what may be at the root of the charge transfer revealed by the Bader charges is the difference in averaged electronegativities between ZnO and TiO_2_; whether calculated in the Pauling or Allred–Rochow scales [42] and with arithmetic or geometric means, it is evaluated to be 0.15–0.3 units higher for TiO_2_ than for ZnO. This explains that there may be some transfer of electronic density from ZnO to TiO_2_ when joining both materials, so that the electronic levels of TiO_2_ are raised; this of course cannot be taken into account in the slabs-versus-vacuum method of calculating band offsets.

Finally, it is worth verifying if the distortions induced in the alternating slab model lead to a significant change in the bandgaps of these materials. For this, distorted bulk structures were obtained from the centre of the fully relaxed alternating slabs, and their electronic structures were evaluated keeping the same cell dimensions and atomic coordinates deduced from these central regions and using the same α values as for the bulk phases. Bandgaps of 3.17 and 3.24 eV were obtained for anatase and ZnO, respectively, indicating that the distortions, while slightly decreasing the bandgaps (by 30 meV in both cases), are probably not enough to reverse the sign of the band offsets.

In summary, any comparison between the results of both models, slabs versus vacuum and alternating slabs, may require a very detailed discussion. In the rest of this paper, other semiconductor pairs will be addressed; only the results similar to those of Figure 4 will be presented in the main text, while the structures and potential or electron density profiles will always be given in the Supplementary Materials.

### 3.2. ZnS-CuGaS_2_ Interface

ZnS and CuGaS_2_ have bandgaps of 3.54 and 2.38 eV, respectively. Both materials have their atoms in tetrahedral coordination, the first one with a fcc arrangement and the second one with a tetragonal chalcopyrite structure; in both cases their (110) planes are nonpolar. According to experimental data, the lattice dimensions of these planes (doubled in the ZnS case) are 7.65 Å × 10.82 Å and 7.58 Å × 10.49 Å, respectively. The misfit is 3% or smaller; one can therefore consider studying interfaces built from these planes.

Their bulk structures were relaxed with the PBE functional, keeping in the valence space 6, 11, 12 and 3 electrons for S, Cu, Zn and Ga, respectively. Relaxing ZnS leads to dimensions in these planes of 7.704 and 10.895 Å; its experimental bandgap is obtained with the HSE06 hybrid functional using α = 0.307. Relaxing equally CuGaS_2_ leads to (110) plane dimensions of 7.582 and 10.622 Å; in this case the experimental bandgap is obtained with HSE06 using α = 0.315. The very similar α values here could be expected since the CuGaS_2_ structure can be derived from that of ZnS simply by changing the neighbours closest to Zn in the periodic table. The structures seen parallel to the (110) planes, as well as the Hartree profiles including the positions of the bands relative to them as deduced from the mentioned HSE06 calculations, are presented for both materials in Figure S1 of the Supplementary Materials.

The structures chosen for the slab/vacuum model include 10 cation layers for each material; thanks to the centred symmetry of both structures the number of atoms can be halved. Figure S2 presents the structures of these slabs once relaxed in atomic positions (not in cell size), together with the Hartree potential profiles and the positions of the VB and CB transferred from the bulk results given in Figure S1. As in Figure 2 above, this allows for the determining of the band offsets within the slab/vacuum model.

The alternating slab model including both materials, with the same amount of cation planes, was built and relaxed as in case I above. The resulting lateral dimensions, as expected, were intermediate between those of the individual phases. The Hartree potential profile was obtained and the positions of the VB and CB were transferred so that the band offsets deduced from this model can be obtained. Figure S3 presents those data.

**Figure 7:**
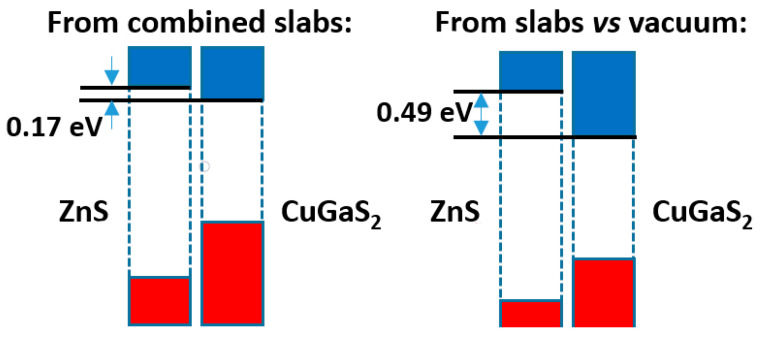
Band offsets for the ZnS|CuGaS_2_ interface.

The comparison of both models is presented in Figure 7. Here, we see that the band offsets, although always with the same sign (leading to a type II band alignment), are more different than in Figure 4 above, i.e., the difference amounts to ca. 0.3 eV. Again, it is worthwhile to try to understand the reason why. As before, the differences of the Hartree potential profiles give no clue, due to the spill of the electronic density towards the vacuum. One may then look at the distribution of electronic density. In this case the density difference curve, presented in Figure S4, does not reveal any significant electron density transfer (its integral up to the minimum of total density amounts only to 0.0002 e^−^/Å^2^); and no dipole seems to have developed. One explanation for the difference between both methods appears when one looks at the Bader charges of the different atoms; they reveal that there is a net transfer of 0.001 e^−^/Å^2^ from ZnS to CuGaS_2_, which may explain at least in part the rise in the levels of the latter material when the two phases are put in contact. Additionally, the averaged electronegativities of CuGaS_2_ are higher by ca. 0.1 units than those of ZnS, which, together with the probably higher softness of (at least) the anions, explains again that some electronic density may have been transferred from ZnS to CuGaS_2_.

### 3.3. A Related System: The Interface between CdS and CuGaS_2_

CdS has a bandgap of 2.3 eV, and since its structure is the same as that of ZnS the (110) surface is again nonpolar. Due to the larger size of Cd the dimensions of that plane, which will be used again to build the interface, are now (once doubled) 8.248 × 11.664 Å, i.e., ca. 9% and 11% larger than those of CuGaS_2_; this may allow to see to what extent the distortions induced when forming the interface in the mixed slab method may alter the results.

CdS, with 12 electrons kept in the valence space of Cd, gave after relaxation at the PBE level a cubic lattice constant of 5.933 Å, implying lateral dimensions of 8.39 × 11.866 Å for the doubled (110) surface. Its bandgap could be reproduced with the HSE06 functional using α = 0.30. Figure S5 presents the Hartree potential profile, together with the positions of VB and CB provided by VASP; the figure given in Section 2.2 for CuGaS_2_ is reproduced here as well, to ease the comparisons. The Hartree potential profile found for both phases within the slab/vacuum model using the same scheme as above is presented, as well as the positions of VB and CB, in Figure S6; this, together with the previous results found for CuGaS_2_, allows obtaining the band offsets within this model.

The alternating slab model was also undertaken; after full relaxation of the structure at the PBE level the lateral dimensions were 7.95 × 11.04 Å, implying significant distortions of these phases. In any case the joint Hartree profile was obtained; it is presented in Figure S7 together with the positions of the VB and CB of both phases as deduced from the hybrid functional calculations of the bulk solids.

This would allow the comparison of the results of both models. However here, due to the larger epitaxial misfit and the ensuing lattice distortions, it is convenient to verify whether there is a change in the positions of the VB and CB which may affect to the band alignments. One way to check this is to build (as said in the anatase|ZnO case), from the innermost regions of both alternating slabs, bulk structures with the same cell periodicity and atom positions of these inner regions, of course with no relaxation at all; and then carry out HSE06 calculations for these distorted bulk phases using the same α value as for the fully relaxed bulks. The result of that calculation, including the Hartree potential profile as well as the band positions, is given in Figure S8a). One can see that the bandgaps have decreased significantly, especially for CuGaS_2_, for which the distorted structure leads to a bandgap even lower than 2.0 eV. If these new band positions are translated to the same Hartree potential profile already obtained for the alternating slab model, the result is given in Figure S8b).

**Figure 8:**
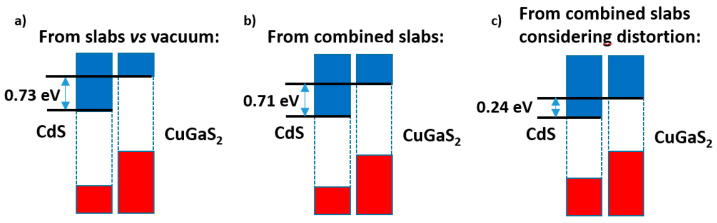
Band offsets found for the CdS|CuGaS_2_ case: (**a**) in the slabs versus vacuum method; (**b**) in the combined slab method, if the lattice distortions are disregarded; (**c**) with these latter taken into account.

Figure 8 presents a summary of these results. While the slabs versus vacuum model (Figure 8a) gives nearly the same band offset as the alternating slabs model when the distortions are disregarded (Figure 8b), once the latter are taken into account (Figure 8c) the band alignment is severely altered; although a type II band alignment still appears, the band offset is reduced by ca. 0.5 eV.

Of course, these distortions will never happen in the real world. Interfaces implying the (110) planes of both materials may indeed occur, but the two phases will try to develop the lattice constants producing in all cases the energy minima; this will result in any type of defects (e.g., stacking faults) which may allow those minima to be approached. For such systems one might study with important cell misfit, as in the CdS|CuGaS_2_ case presented here, the electron density distribution in the mixed slabs model, as well as the corresponding electron density transfer; but no useful information may be gained from this, as these distortions will never be real. Even the result of the Bader analysis, yielding here a (rather small) transfer of atomic charge from CdS to CuGaS_2_ of 0.0005 e^−^/Å^2^, which is in agreement again with the prediction considering the averaged electronegativities, must be used with caution.

In the end, the most convenient way of handling a problem such as the one found here may consist in developing larger supercells in the directions parallel to the desired interface which allow for a smaller overall strain, even if this implies a larger number of defects or unsatisfied bonds at the interface; this was performed for example in ref. [43]. Indeed, the contacting phases, especially if they are not present as ultrathin films, will manage to grow in such a way that their bandgaps are not disturbed much from the natural ones. This may take place by contact through different crystal planes, developing stacking faults (or other defect types) and accumulating impurities at the interface, etc.

### 3.4. The Rutile TiO_2_-PbTe Interface

Rutile-type TiO_2_ has a bandgap of 3.0 eV, much larger than that of PbTe (0.27 eV [44]). TiO_2_, with tetragonal structure, has a = b = 4.594 Å and c = 2.959 Å; PbTe, when the symmetry of its fcc structure is decreased to centred tetragonal, has a = b = 4.569 Å. Since both (001) planes are nonpolar (although it is well known that for rutile TiO_2_ the most stable surface is the (110) one), they provide a nice example of near-perfect epitaxy (the misfit is ~0.5%).

These structures were relaxed with the PBE functional, keeping in the valence space 6, 10, 14 and 24 electrons for O, Ti, Pb and Te, respectively. In the case of PbTe it is necessary to include the spin-orbit relativistic interaction, due to the especially heavy nature of the Pb cation. The fully relaxed PbTe, including the spin-orbit coupling (henceforth mentioned as S.-O. coupling), has (in the tetragonal setting) a = 4.627 Å; its bandgap is reproduced with HSE06 functional using α = 0.115. Here it is found that, if the S.-O. coupling is not included, the hybrid calculation with the same α value yields a gap of 1.00 eV. The situation is thus similar to that well known in the case of the nowadays much studied methylammonium lead iodide perovskite, which also includes divalent Pb; in that case, neglect of the S.-O. coupling leads to a bandgap 0.9 eV higher [45].

In order to be consistent, one also has to apply the spin-orbit interaction to the rutile case (although doing so negligibly changes its bandgap), as well as to all the other calculations in this section. After the PBE relaxation (including S.-O. coupling), rutile TiO_2_ has a = 4.601 Å and c = 2.953 Å. Its bandgap can be reproduced with HSE06 functional (including again S.-O. coupling) using α = 0.21. The resulting Hartree potential profiles for both semiconductors in the direction perpendicular to the (001) plane are given in Figure S9, including also the positions of VB and CB relative to them.

As in the preceding cases, Figure S10 presents the Hartree potential profiles of the TiO_2_ and PbTe (001) slabs contiguous to the vacuum, relaxed in atomic coordinates but with the dimensions parallel to the surface fixed to the bulk values; the positions of the VB and CB relative to them, as transferred from the respective hybrid calculations carried out for the bulk phases, are also included, so that the band offsets resulting from this model can be obtained. Note that in this case the number of cation planes of the rutile phase (17) had to be significantly higher than that chosen for the PbTe phase (9); not because of the shorter interplanar spacing in the first case, but mainly due to the oscillating nature of the Hartree profile found for the rutile, which required ensuring a converged enough profile in this case.

For the alternating slabs model, the resulting Hartree potential profile, again with the same number of cation layers, is presented in Figure S11 together with the corresponding positions of the VBs and CBs. The band offsets resulting from this model can thus be estimated.

**Figure 9:**
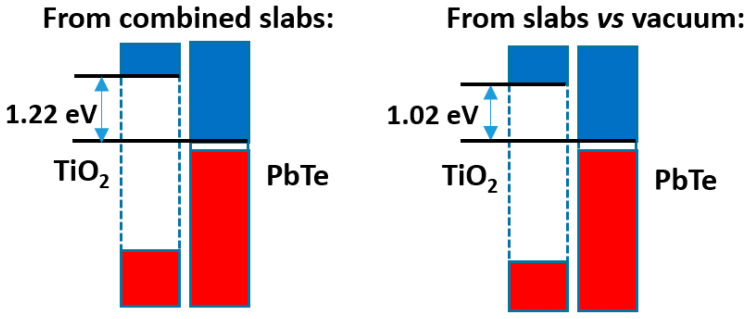
Band offsets found for the rutile TiO_2_|PbTe case.

The comparison of both models is presented in Figure 9. It can be seen that the difference in band offsets is ca. 0.20 eV. This is clearly smaller than the band offset itself (over 1.00 eV), but is not insignificant. As in the preceding cases, the total electronic density distribution is almost the same as the sum of the densities of the separated slabs; the relevant quantity is the difference in electron densities, which is presented in Figure S12 together with the full electron density profile of the compound slab. This difference is not really large, and amounts, up to the point of minimum density between slabs, to a transfer from PbTe to TiO_2_ of 0.002 e^−^/Å^2^. There is some hint of a dipole at both sides of the interface, with higher amount of electronic density, in both sides, towards the side of PbTe. If one looks at the Bader charges, it can be seen however that that the amount of charge transferred from PbTe to TiO_2_ is 0.0086 e^−^/Å^2^. This agrees with the difference in electronegativity of both materials, which is higher in TiO_2_ by 0.6–1.0 units (depending on the way it is estimated).

### 3.5. The Interface between the (111) Planes of Diamond and α-Tin

C in the diamond form and the semiconducting α-tin form (also called gray tin) have cubic fcc structures with a = 3.567 and 6.489 Å, respectively. The ratio between these values is 1.819, thus differing only 5% from $\sqrt{3}$ = 1.732. This implies that a $\sqrt{3}\times \sqrt{3}$ supercell of the (111) plane in diamond, with a = 4.368 Å, can be combined with the (111) plane of α-tin (where a = 4.588 Å). Their bandgaps are certainly very different: for diamond the bandgap is 5.44 eV, while for α-tin it is only ~0.1 eV [46].

The structures were relaxed with the PBE functional, keeping in the valence space 4 and 14 electrons for C and Sn, respectively. For the Sn primitive cell this resulted in a = 4.707 Å for the (111) surface (therefore a = 6.656 Å for the conventional cell), and its bandgap could be reproduced with the HSE06 functional using α = 0.20. It was verified that including the spin-orbit coupling, together with this α value, did not lead to a significant change in the bandgap. For the primitive lattice of diamond-type C this relaxation resulted in a = 2.527 Å (therefore a = 3.574 Å for the conventional cell), so that $\sqrt{3}$ times this value implies 4.377 Å; its bandgap could be reproduced with HSE06 using α = 0.27. For both cases the Hartree potential profiles in the direction perpendicular to the (111) plane were obtained; they are presented, together with the positions of the VB and CB, in Figure S13.

When working with the slabs, care was taken in this case to use spin-polarized calculations, since one should take into account, especially for diamond, the possibility of having dangling bonds containing unpaired electrons. For both materials, slabs facing the vacuum with interfaces parallel to the (111) plane and containing nine planes, having the lateral dimensions fixed at the values of the bulk, were built and relaxed in the internal atomic coordinates; the resulting Hartree potential profiles are shown in Figure S14 together with the positions of VB and CB as transferred from the results in Figure S13. One may note the significant distortion of the profile for the outermost layer in the α-tin. Additionally, spin densities appear in the C slab joining vacuum: the values projected inside the PAW sphere amount to 0.45 for those C atoms presenting dangling bonds, and there are also values of 0.12 in some deeper C atoms. This is compatible with the minimum gap computed here at the PBE level for this slab: 2.06 eV, indicating that a metallic character is not present. In the Sn slab facing the vacuum, however, no net spin density is found for any atom, not even those which might have dangling bonds. Probably, although such bonds may be present, a metallic behaviour occurs in this case, with equal population of up and down spins in all atoms. However, these results probably do not reflect the real experimental situation; see below the comments on known reconstructions of these surfaces.

The structure of the completely relaxed compound slab including the same number of atomic layers is presented in Figure S15, together with the Hartree potential profile and the corresponding positions of the VB and CB. Here, however, no spin density is found on any atom; this might be due to the metallic character of the Sn-C interface, which affects the overall spin density distribution equaling for all atoms the population of up and down spins. Note also that the distortion of atomic positions revealed in Figure S13 is not present here.

**Figure 10:**
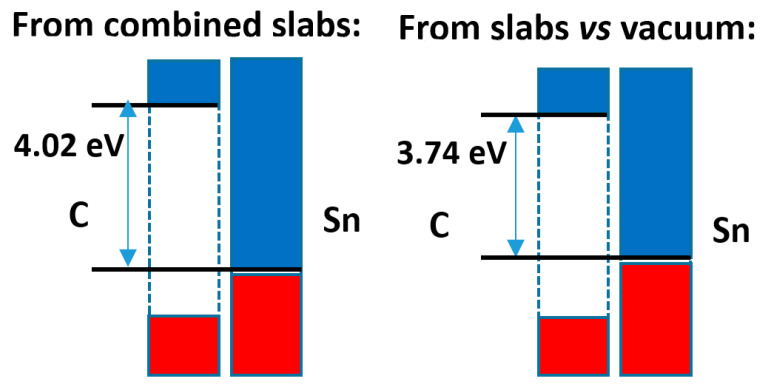
Band offsets found for the diamond-type C|α-tin case.

The comparison of both calculation methods is presented in Figure 10. Here, the difference in band offsets is higher, almost 0.3 eV, although this does not alter the character of the band alignment that remains of type 1. The analysis of the electron density distribution, shown in Figure S16, indicates an electron density transfer from Sn to C of 0.006 e^−^/Å^2^, while the Bader analysis indicates that there is a relatively large transfer of electrons from Sn to C of 0.06 e^−^/Å^2^, in agreement with the difference of average electronegativities (ca. 0.6/0.8 units in the Pauling/Allred–Rochow scales, respectively).

However, it turns out that for diamond-type C it was reported that a 2 × 1 reconstruction exists for the (111) surface [47]. For Sn (111), two reconstructions are known (at least for 50 ML thin films): a (3 × 3) one existing at ambient temperature and a (2 × 2) one appearing in the interval between 50 and 150 °C, being followed at higher temperature by a (1 × 1) structure, shortly before fusion [48]. All these reconstructions, of course, were identified for interfaces between these materials and vacuum. It makes no sense trying to study them, since what one wants to study here is the band offset appearing when both materials are joined, and due to the electronegativity difference one can foresee that a significant electron density transfer from Sn to C will then appear. The main issue here then is that one cannot know which reconstructions might arise at an interface between Sn and C (111) surfaces; they might be quite different from those experimentally observed for the individual surfaces.

## 4. Conclusions

The comparison of both methods of band offset estimation (alternating slabs of both materials and individual slabs/vacuum interfaces) shows that differences in band offsets of (at least) up to 0.3 eV may appear among them (but note that the result in Figure 8b is incorrect). In many cases this might not affect the overall picture, but at least in one case (the anatase|ZnO interface) it was found that the relative positions of the conduction bands may be reversed, which may be relevant for the prediction of the direction of the electron transfer.

That said, one must remember that there are several cautions that must be exercised. Firstly, one must ensure that bandgaps are reproduced accurately for both materials; this almost certainly requires the use of hybrid functionals, probably with different fractions of Fock exchange for each material. Of course, nonpolar surfaces with symmetry-equivalent interfaces, must be used throughout, otherwise the relationship of the band edges to the electrostatic (Hartree) potential profile (the method used here throughout, and also by many authors) can be very uncertain. One must pay attention also to the possibility that the individual surfaces facing the vacuum may show reconstruction in larger supercells; if so, unless experimental data are available, there is no certainty that one or other of these reconstructions occurs in the combined interface.

Then there are other factors. On the one hand, the method of surfaces versus vacuum may have the disadvantage that the spill of electronic density towards the vacuum will interfere with an exact calculation. In any case if the materials have a significant difference in average electronegativity the foreseeable electron density transfer between them will never be captured by that method. Therefore, the method of alternating slabs will always be preferred, and will include the electronic charge transfer due to average electronegativity differences as captured by the Bader charges of each slab, unless there is a significant epitaxial mismatch between both phases. In this last case, the consequences can only be monitored verifying whether, capturing from the centre of each of both phases each distorted geometry, there is a significant change in the resulting bandgap and in the positions of the bands with respect to the Hartree potential profile of the distorted phases. Then, the only way to solve the problem might be to use larger supercells so that the epitaxial mismatch can be reduced, even if this leads to stacking faults and lower number of well-formed bonds.

Finally, there are another two aspects that have not been taken into account here. On the one hand, no doping of the semiconductors is considered that may make them n- or p-type; if such doping occurs, band bending(s) may appear, due to interfacial charge transfer, which will alter the Fermi energies. Although this doping might not significantly change the positions of the VB and CB themselves, the induced band bending could largely influence the direction and extent of electron and hole flow.

On the other hand, if one or both of the semiconductors is in contact with a polar solvent the band alignment can be modified. This can be exemplified by a relatively recent article by Scanlon et al. [12] which claimed, based on theoretical calculations confirmed by XPS experimental data, that the conduction band of rutile TiO_2_ lies above that of anatase TiO_2_, contradicting the usual belief based on electrochemical data, that the anatase conduction band lies above that of the rutile. The contradiction was solved shortly after in a paper by Kullgren et al. [49] showing, also with theoretical calculations, that the situation can be reversed if the presence of water (and in some cases also of OH^−^ or H^+^ ions), as is obviously the case in most electrochemical measurements, is taken into account, due (at least) to the different surface density of water dipoles adsorbed in both cases. Therefore, if the materials are in contact with a polar solvent, or with any solvent containing electrolytes, it may be that neither the alternating slabs model nor the slabs versus vacuum model can give accurate enough predictions.

## Data Availability

No problem involving ethics is applicable. On the other hand, once this article is published it will be made available at several repositories.

## Supplementary Materials

The following are available online at https://www.mdpi.com/article/10.3390/nano11061581/s1, Figure S1: Structures of ZnS and CuGaS_2_, with the Hartree potential profiles and the positions of the bands marked in both cases; Figure S2: Hartree potentials obtained for slab models of ZnS and CuGaS_2_ facing vacuum, with the positions included of VB and CB (as deduced from the HSE06 calculations of the bulk solids, see Figure S1). Only (less than) half of the unit cell is presented in both cases; Figure S3: Structure of the mixed slab model for the ZnS|CuGaS_2_ interface, with the Hartree potential profile obtained for it and including the positions of the VB and CB deduced from Figure S1; Figure S4: Displaying the total electronic density (within the PAW core representation) for the relaxed mixed slab of ZnS and CuGaS_2_, together with that of the ZnS and CuGaS_2_ individual slabs with same atomic positions; in the lower part, the corresponding charge density difference; Figure S5: Structures of CdS and CuGaS_2_, together with their Hartree potential profiles and the corresponding positions of the VB and CB; Figure S6: Hartree potential profiles for CdS and CuGaS_2_ within the slabs versus vacuum method; Figure S7: Structure of the CdS|CuGaS_2_ interface, and the resulting plane-averaged Hartree potential profile including the position of the VB and CB of both materials as translated from the results given in Figure S5; Figure S8: (a) Hartree potential profiles, and positions of the VB and CB relative to them, for structures of bulk CdS and CuGaS_2_ built from the central regions of the alternating slab structure presented in Figure S7. (b) New positions of the VB and CB of both phases once the results in Figure S8a) are taken into account; Figure S9: Structures of rutile TiO_2_ and PbTe displayed parallel to their (001) planes, together with the respective plane-averaged Hartree profiles and the VB and CB positions computed with HSE06 functionals as explained in the main text; Figure S10: Hartree potential profiles for rutile TiO_2_ and PbTe within the slabs versus vacuum method; Figure S11: Structure of the alternating slabs model of the TiO_2_|PbTe (001) interface, including the plane-averaged Hartree potential profile computed for it and the positions of the VB and CB of both components as transferred from the hybrid calculations for the bulk solids given in Figure 9; Figure S12: Charge density distribution, and its difference with the sum of the individual slab densities (keeping cell and atom positions unchanged) for the rutile TiO_2_|PbTe interface; Figure S13: Structures of diamond-type C and α-tin seen parallel to the (111) plane, and the corresponding Hartree potential profiles; the positions of VB and CB are indicated as well; Figure S14: Hartree potential profiles for slabs of diamond-type C and α-tin facing vacuum, with the positions of VB and CB transferred according to the results in Figure S13; Figure S15: Structure of the diamond-type C and α-tin compound slab, including the Hartree potential profile and the positions relative to it of the VB and CB of both materials; Figure S16: Charge density profile for the diamond-type C|α-tin compound slab, and its difference with the sum of the individual slab densities (keeping cell and atom positions unchanged).
