# Supplementary material for: Computing with DFT Band Offsets at Semiconductor Interfaces: A Comparison of Two Methods
Source: Nanomaterials (Basel). 2021 Jun 16;11(6):1581. doi: 10.3390/nano11061581 (PMC8235794; doi:10.3390/nano11061581)

Computing with DFT Band Offsets at Semiconductor Interfaces: A Comparison of Two Methods

José C. Conesa

Instituto de Catálisis y Petroleoquímica, CSIC, 28049 Madrid, Spain; jcconesa@icp.csic.es

SUPPLEMENTARY MATERIALS

Figure S1 Structures of ZnS and $CuGaS_2$, with the Hartree potential profiles and the positions of the bands marked in both cases.

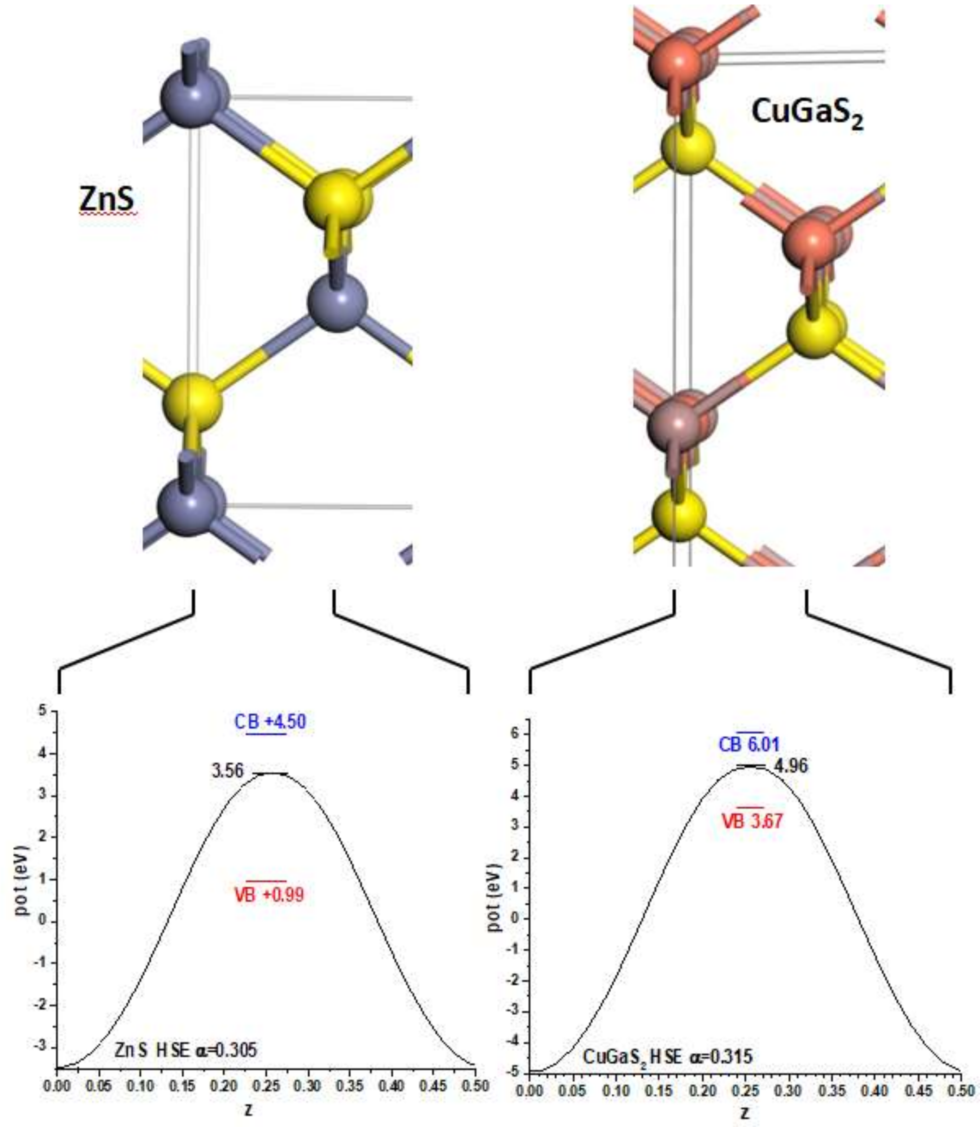

Figure S2 Hartree potentials obtained for slab models of ZnS and $CuGaS_2$ facing vacuum, with the positions included of VB and CB (as deduced from the HSE06 calculations of the bulk solids, see Figure S1). Only (less than) half of the unit cell is presented in both cases.

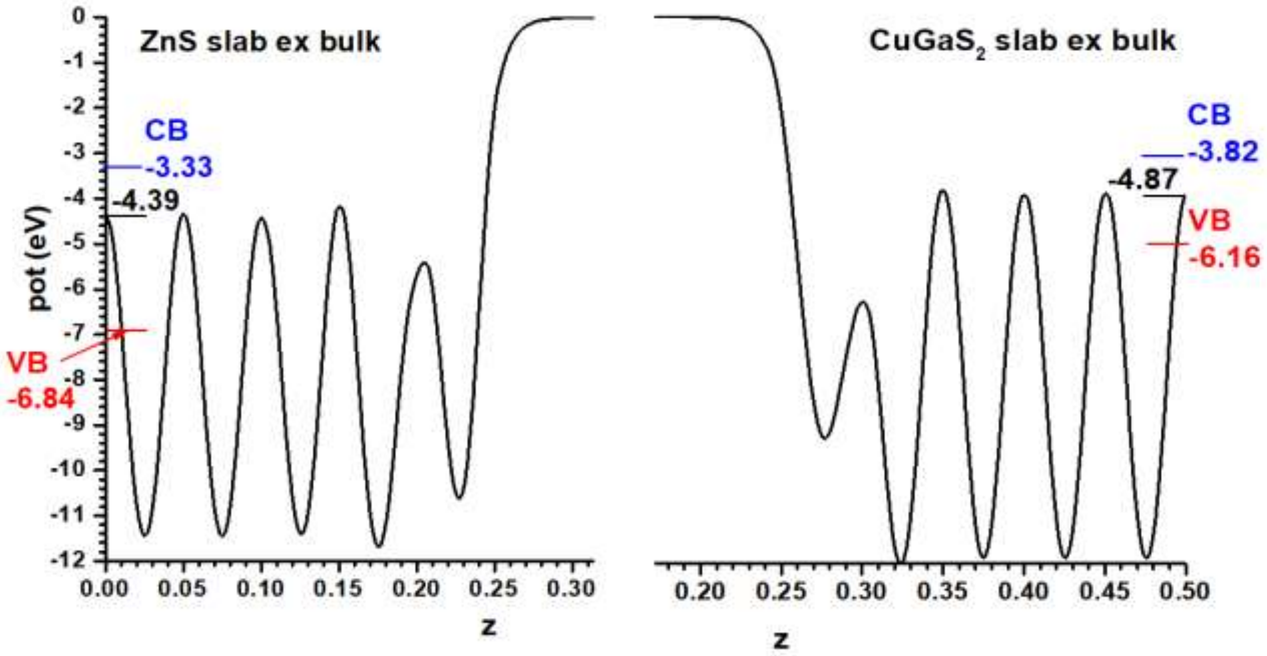

Figure S3 Structure of the mixed slab model for the $ZnS|CuGaS_2$ interface, with the Hartree potential profile obtained for it and including the positions of the VB and CB deduced from Figure S1.

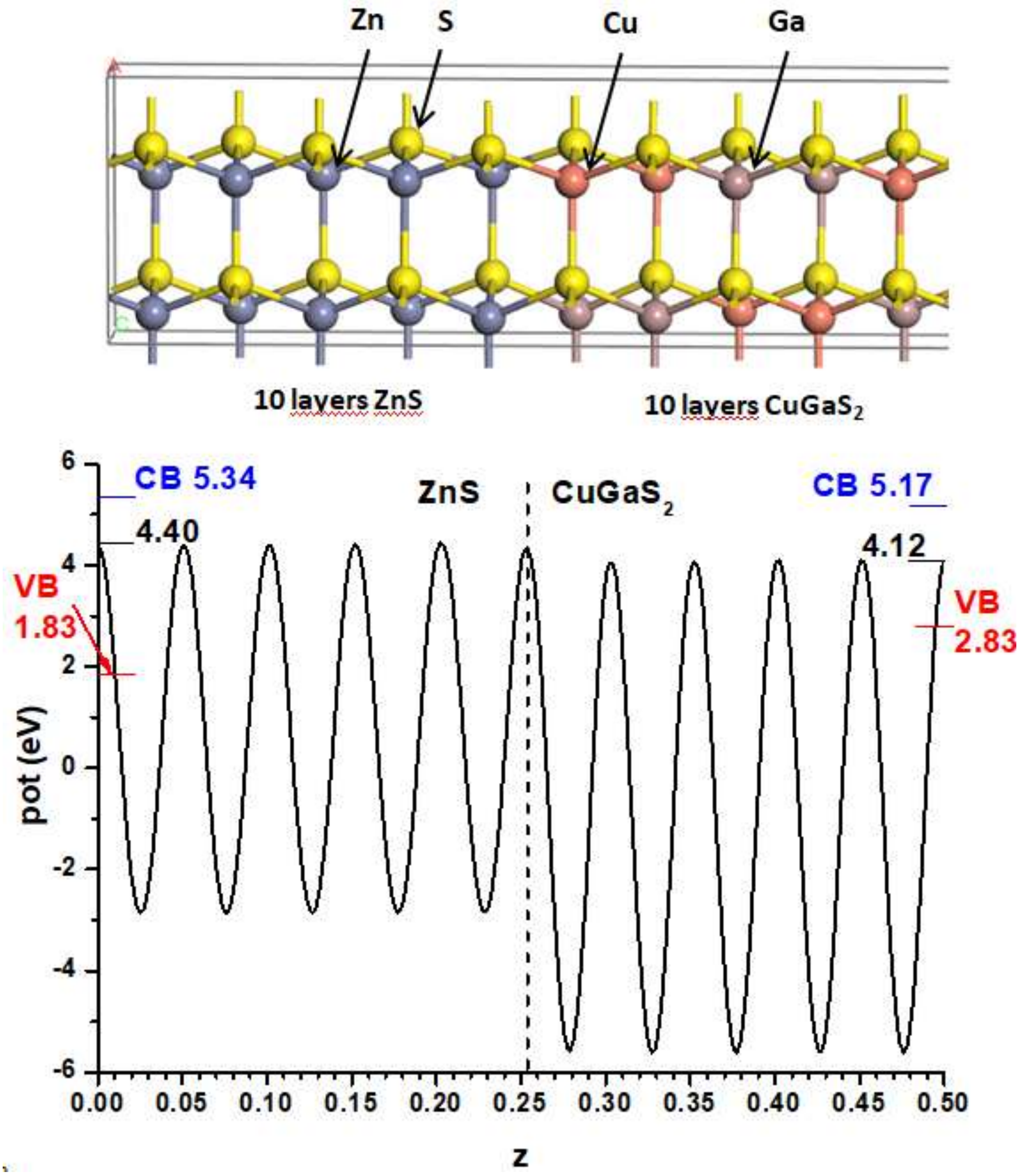

Figure S4 Displaying the total electronic density (within the PAW core representation) for the relaxed mixed slab of ZnS and $CuGaS_2$, together with that of the ZnS and $CuGaS_2$ individual slabs with same atomic positions; in the lower part, the corresponding charge density difference.

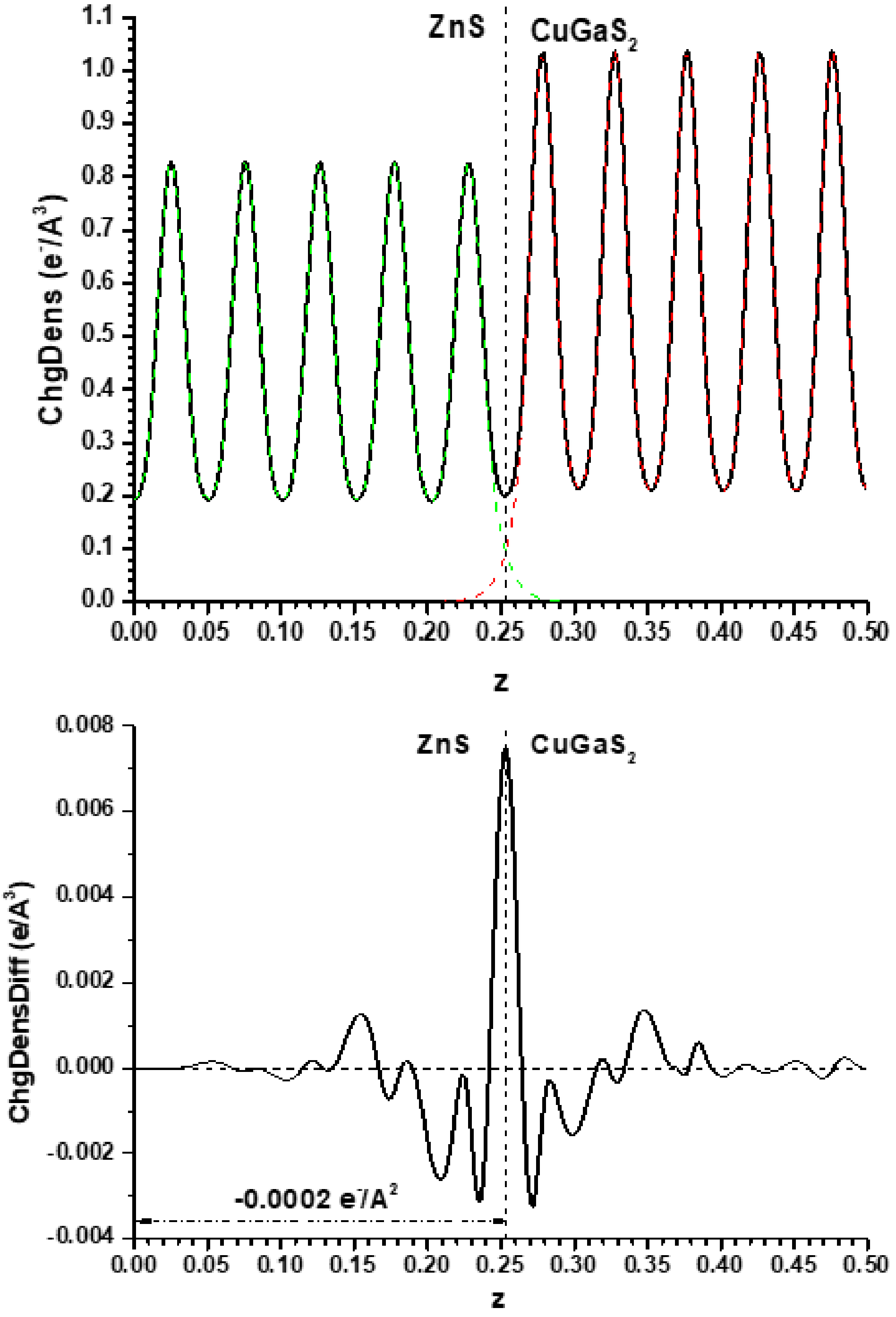

Figure S5 Structures of CdS and $CuGaS_2$, together with their Hartree potential profiles and the corresponding positions of the VB and CB.

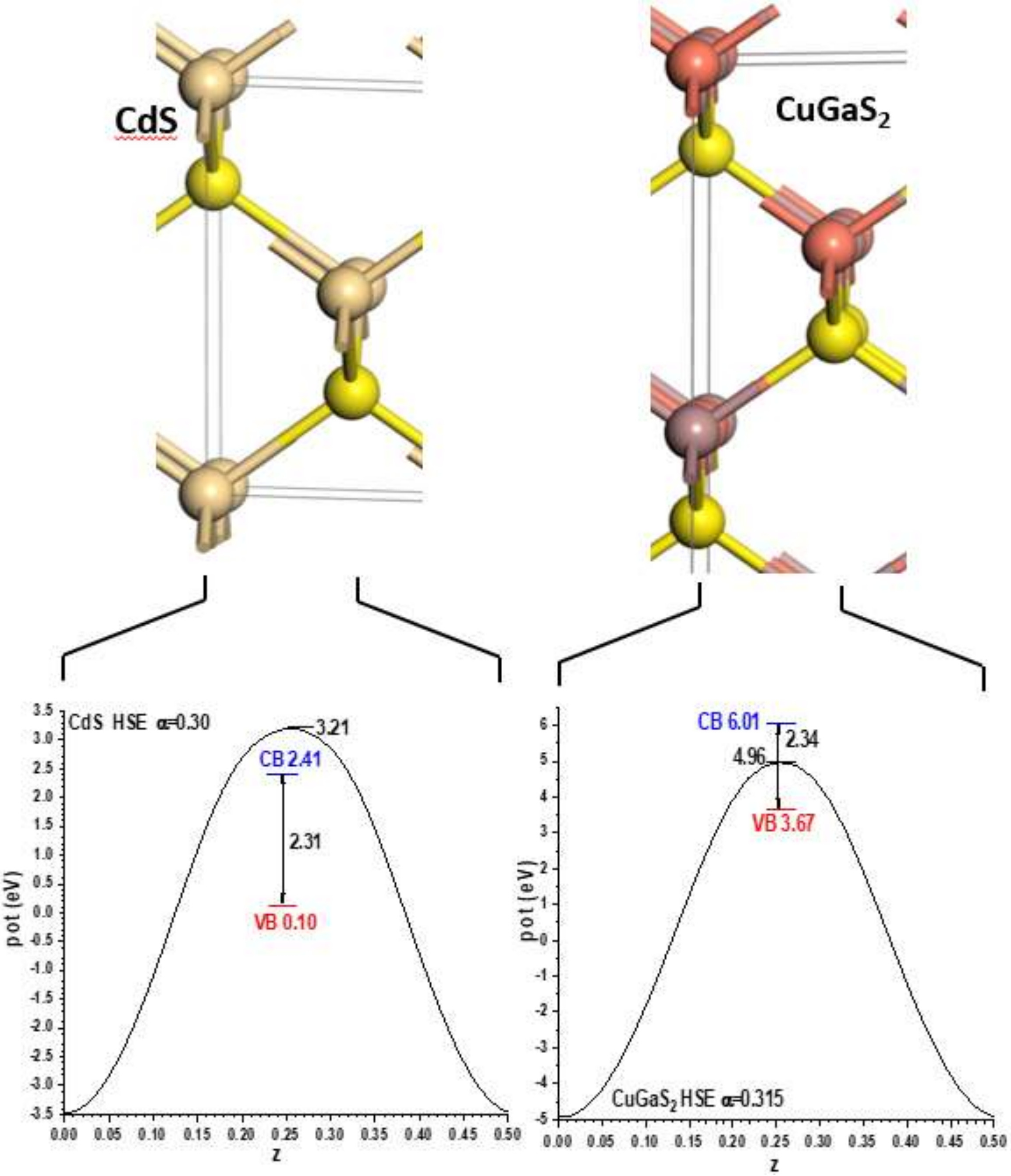

Figure S6 Hartree potential profiles for CdS and $CuGaS_2$ within the slabs versus vacuum method.

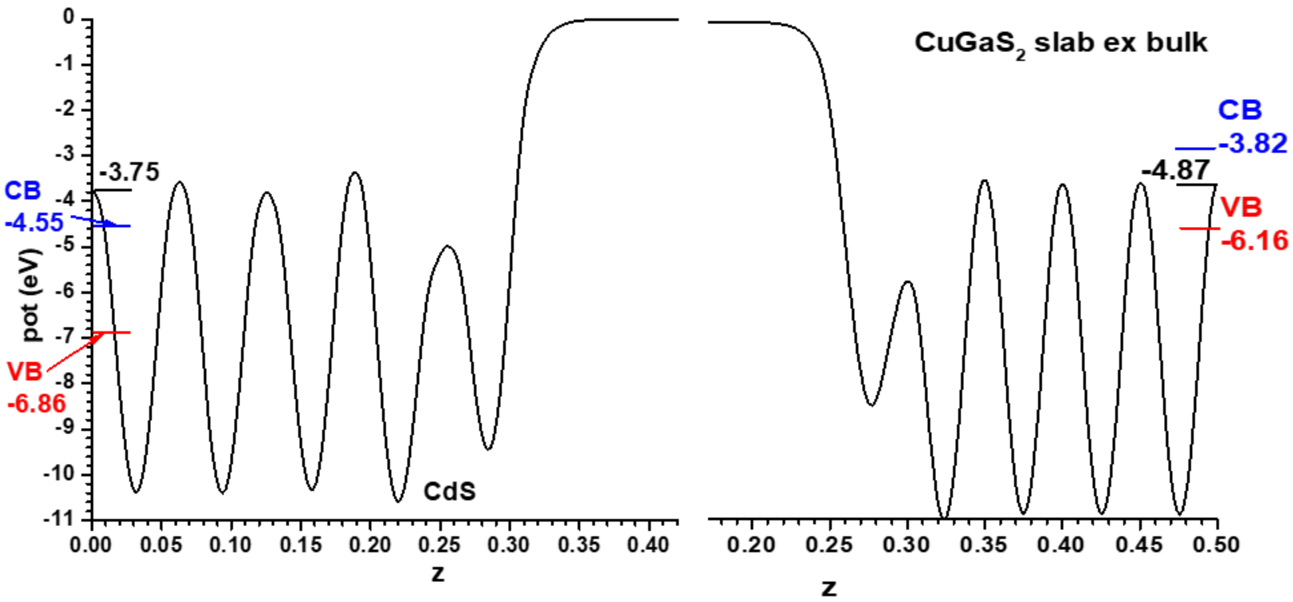

Figure S7 Structure of the $CdS|CuGaS_2$ interface, and the resulting plane-averaged Hartree potential profile including the position of the VB and CB of both materials as translated from the results given in Figure S5.

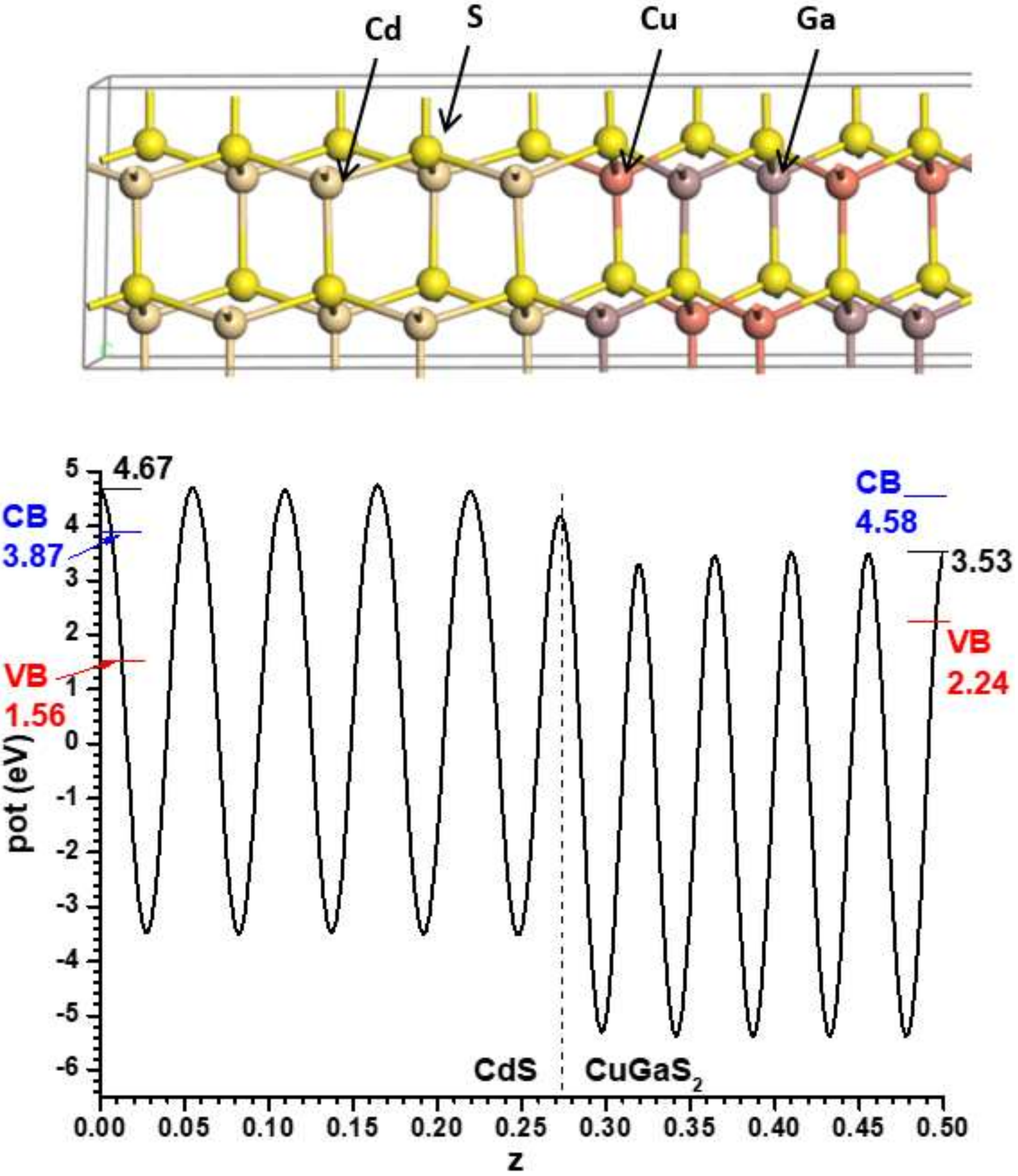

Figure S8 (**a**) Hartree potential profiles, and positions of the VB and CB relative to them, for structures of bulk CdS and $CuGaS_2$ built from the central regions of the alternating slab structure presented in Figure S7. (**b**) New positions of the VB and CB of both phases once the results in Figure S8a) are taken into account.

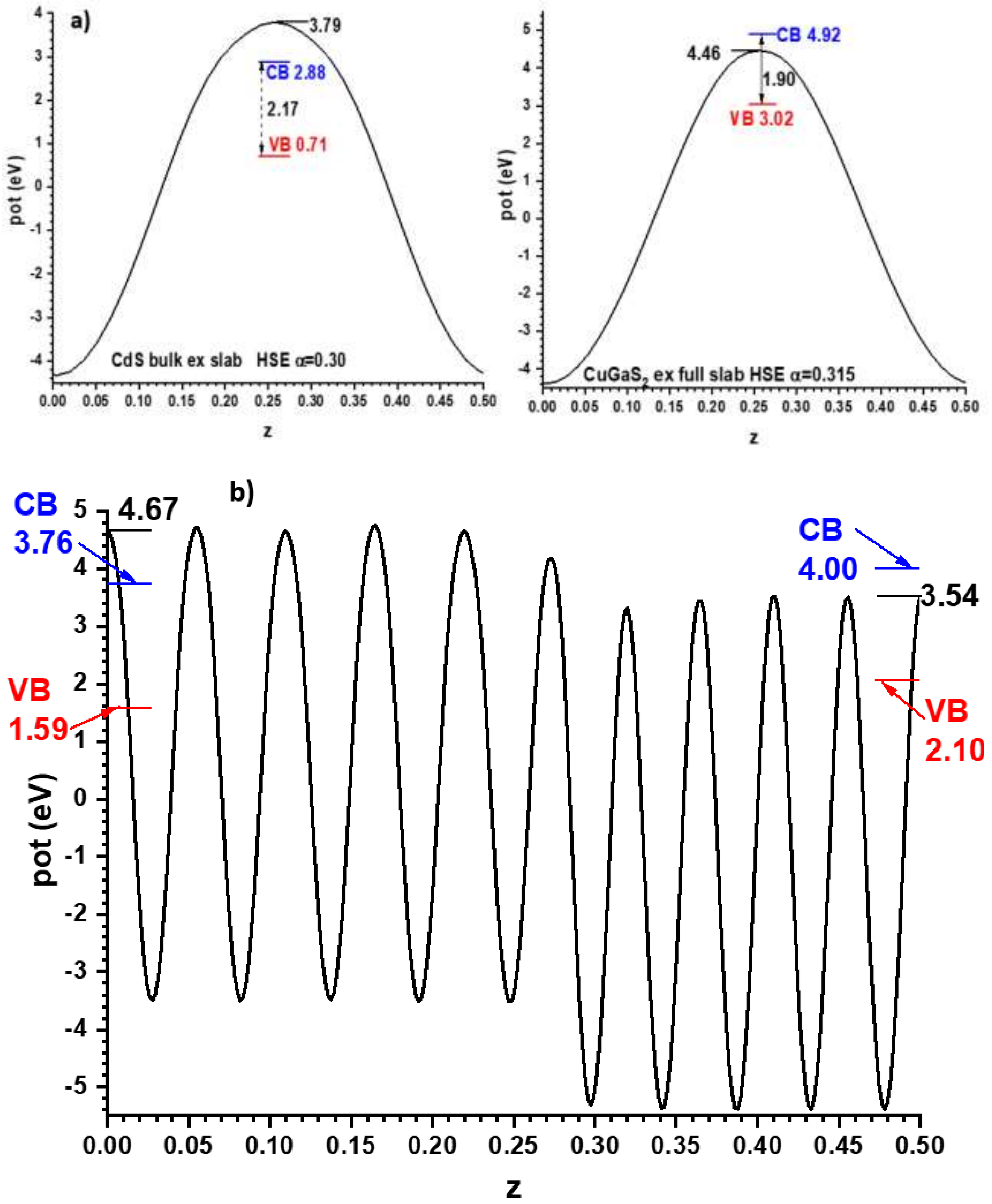

Figure S9. Structures of rutile TiO_2 and PbTe displayed parallel to their (001) planes, together with the respective plane-averaged Hartree profiles and the VB and CB positions computed with HSE06 functionals as explained in the main text.

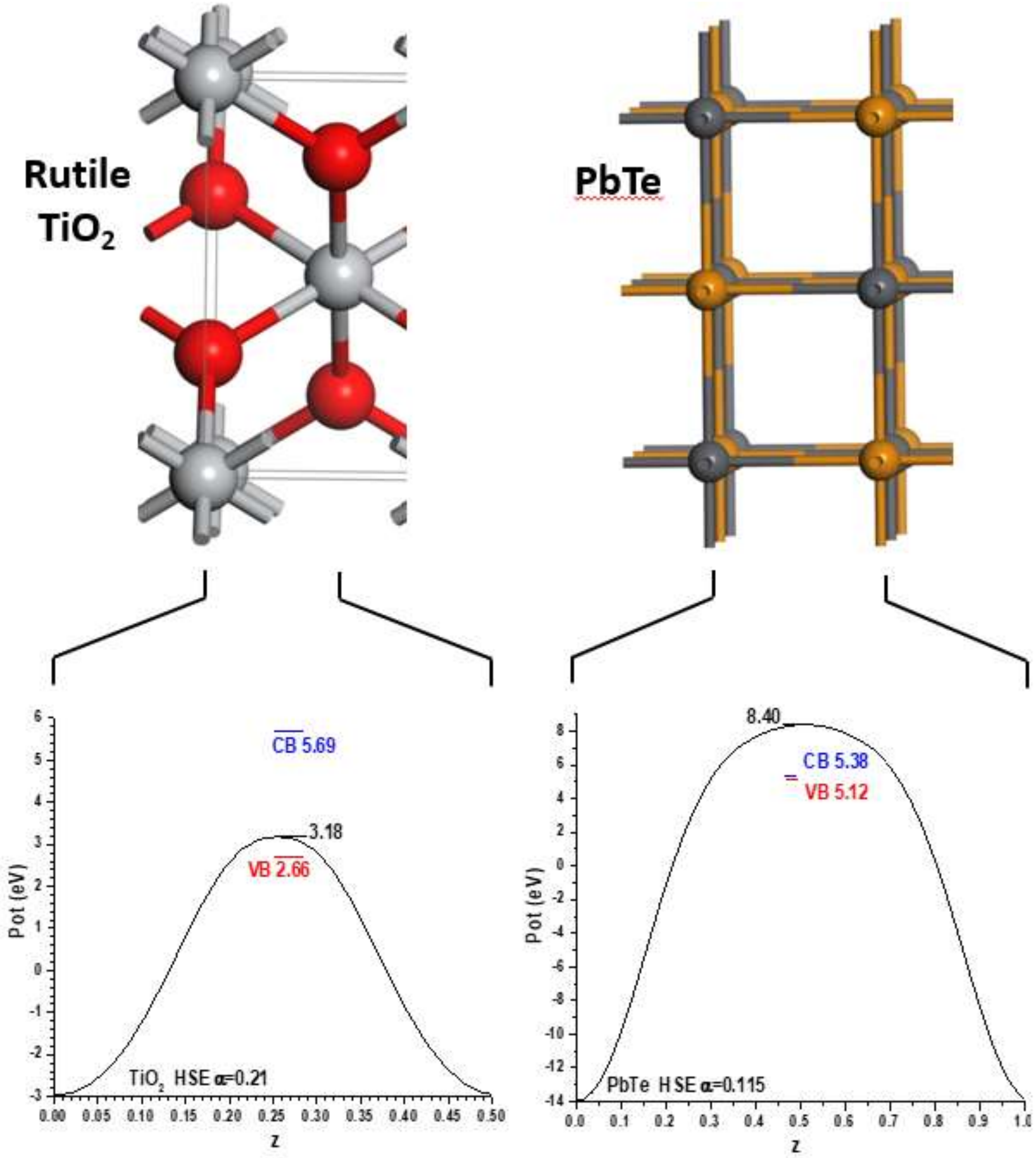

Figure S10 Hartree potential profiles for rutile TiO_2 and PbTe within the slabs versus vacuum method.

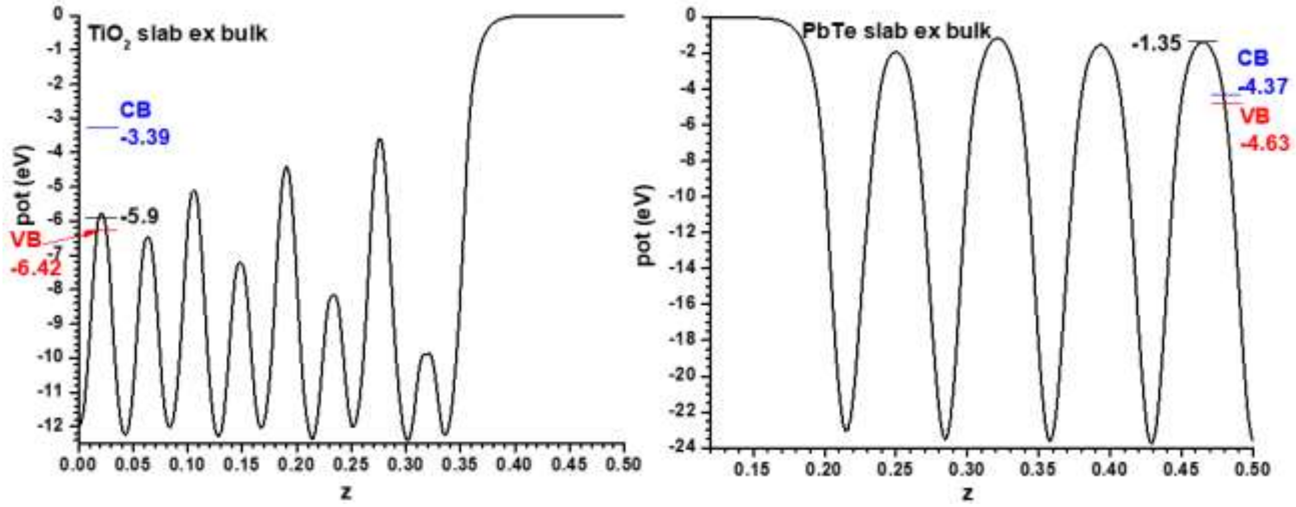

Figure S11 Structure of the alternating slabs model of the TiO_2|PbTe (001) interface, including the plane-averaged Hartree potential profile computed for it and the positions of the VB and CB of both components as transferred from the hybrid calculations for the bulk solids given in Figure 9.

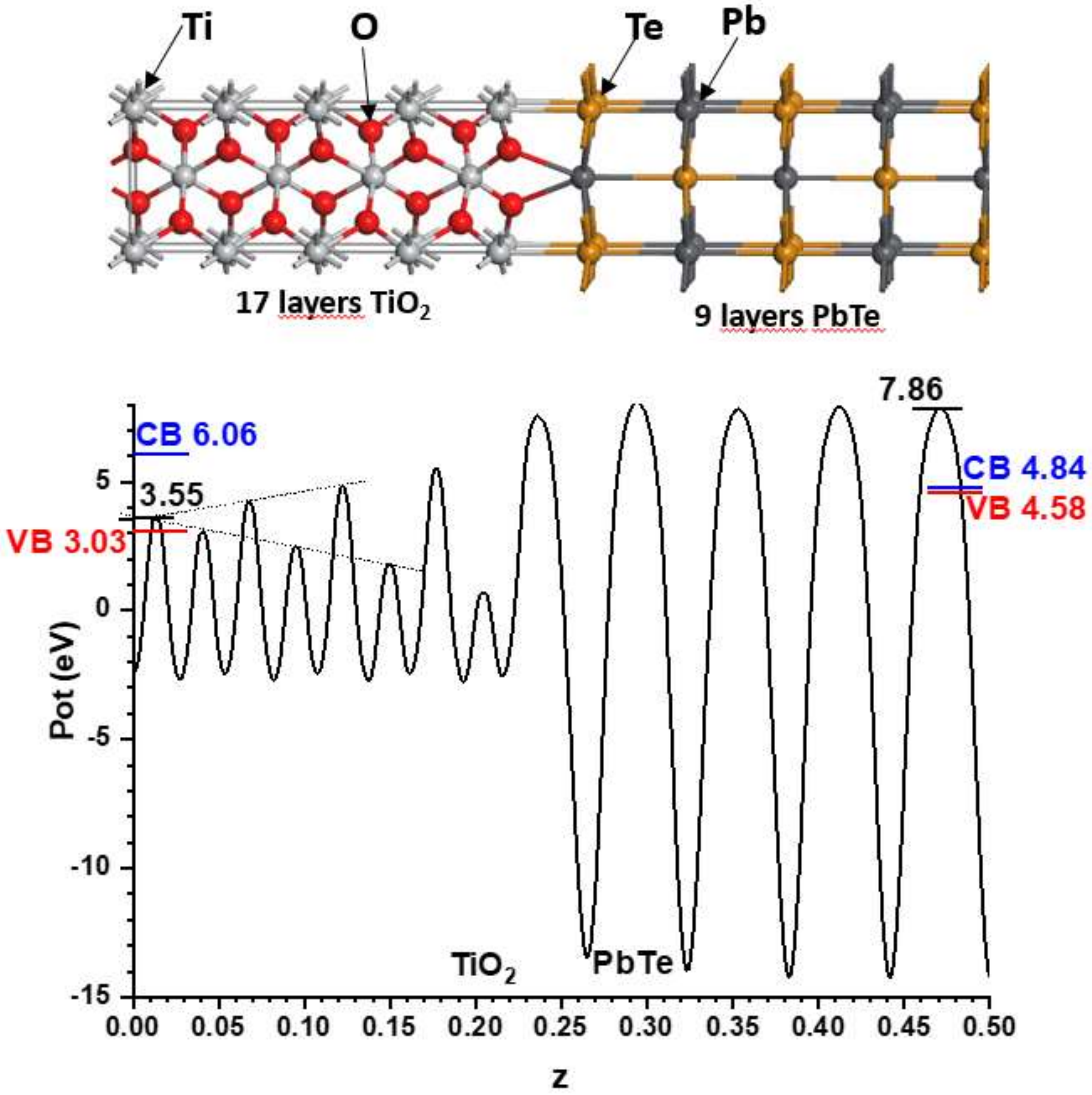

Figure S12 Charge density distribution, and its difference with the sum of the individual slab densities (keeping cell and atom positions unchanged) for the rutile TiO_2|PbTe interface.

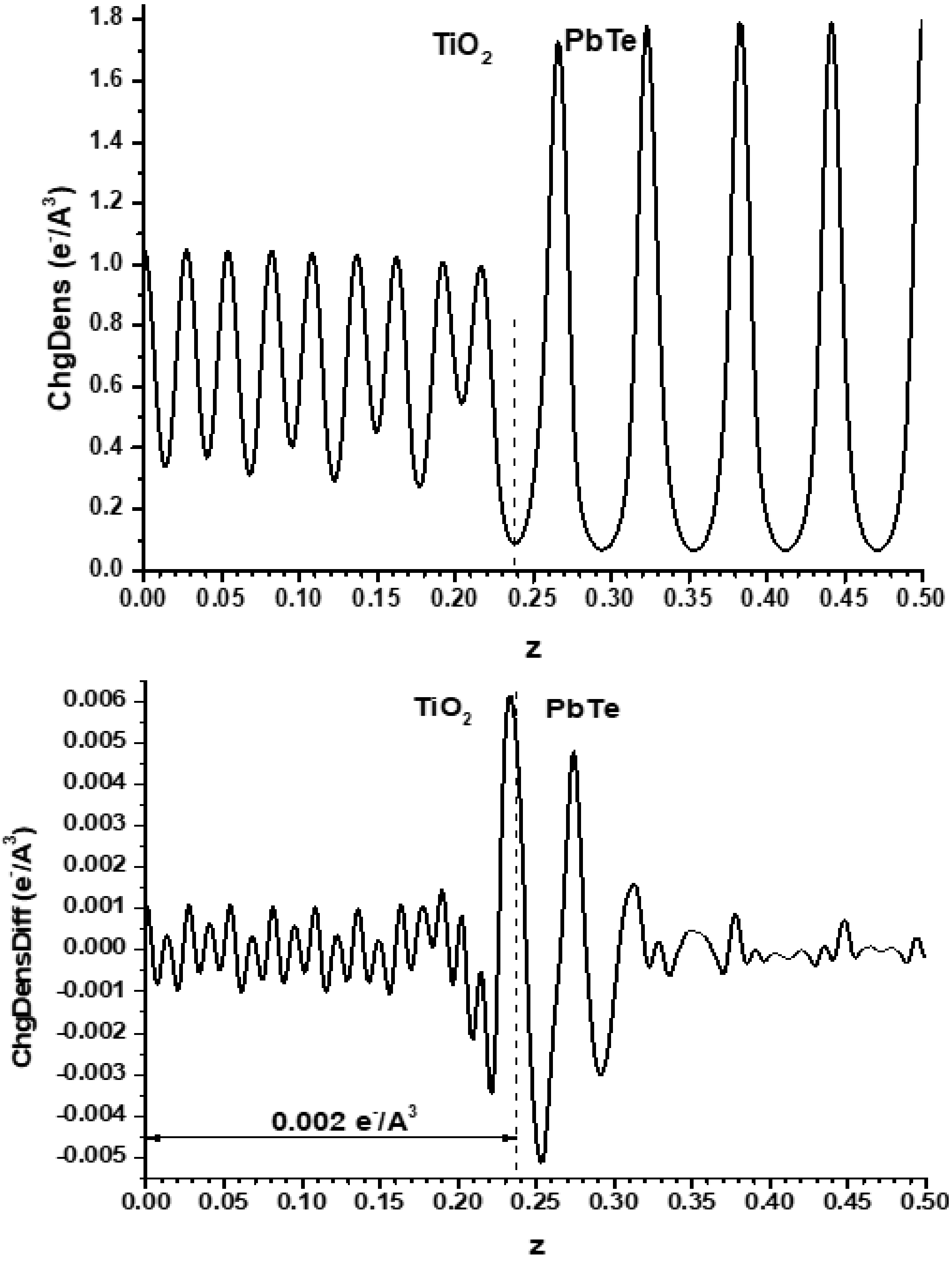

Figure S13 Structures of diamond-type C and α-tin seen parallel to the (111) plane, and the corresponding Hartree potential profiles; the positions of VB and CB are indicated as well.

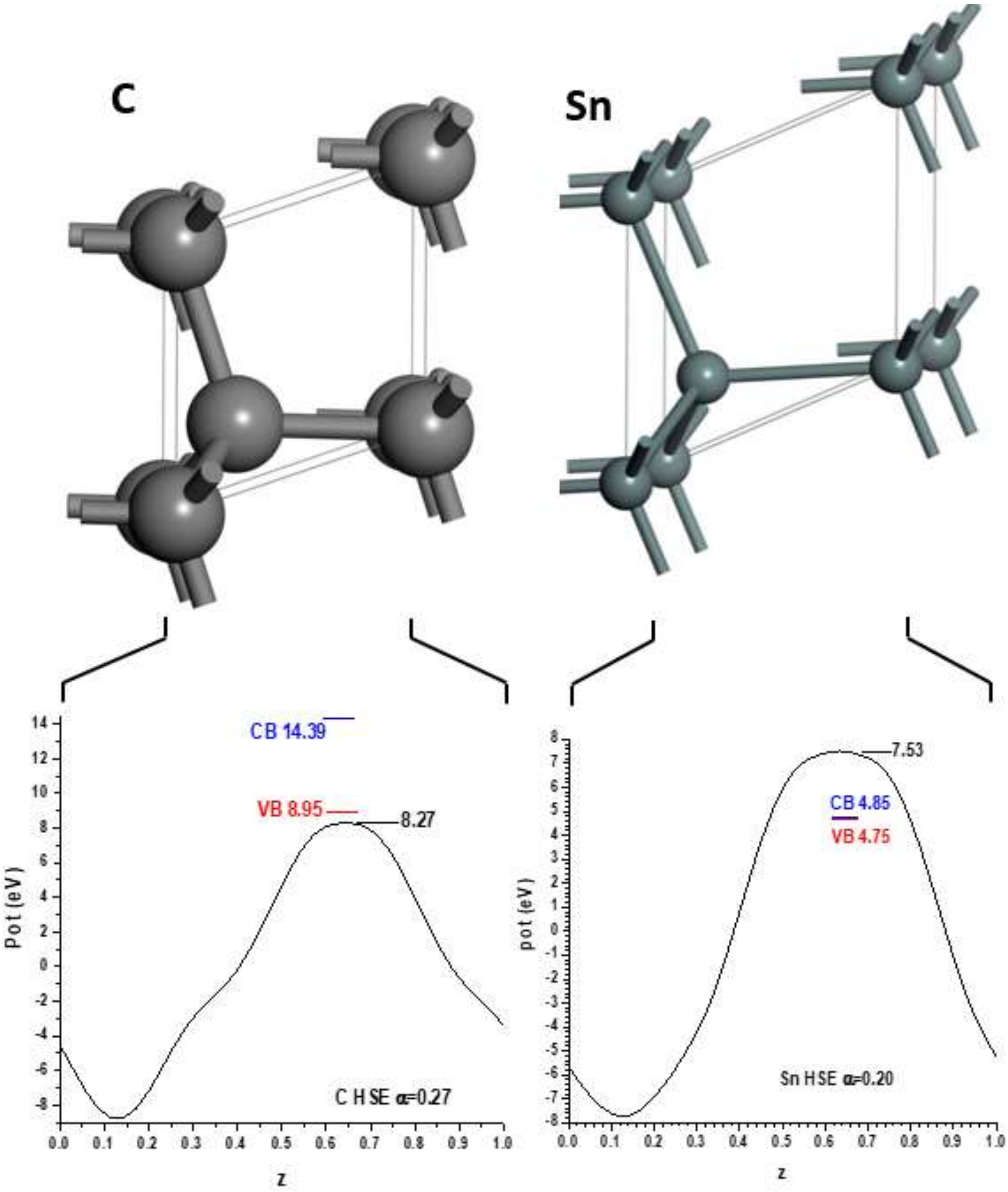

Figure S14 Hartree potential profiles for slabs of diamond-type C and α-tin facing vacuum, with the positions of VB and CB transferred according to the results in Figure S13.

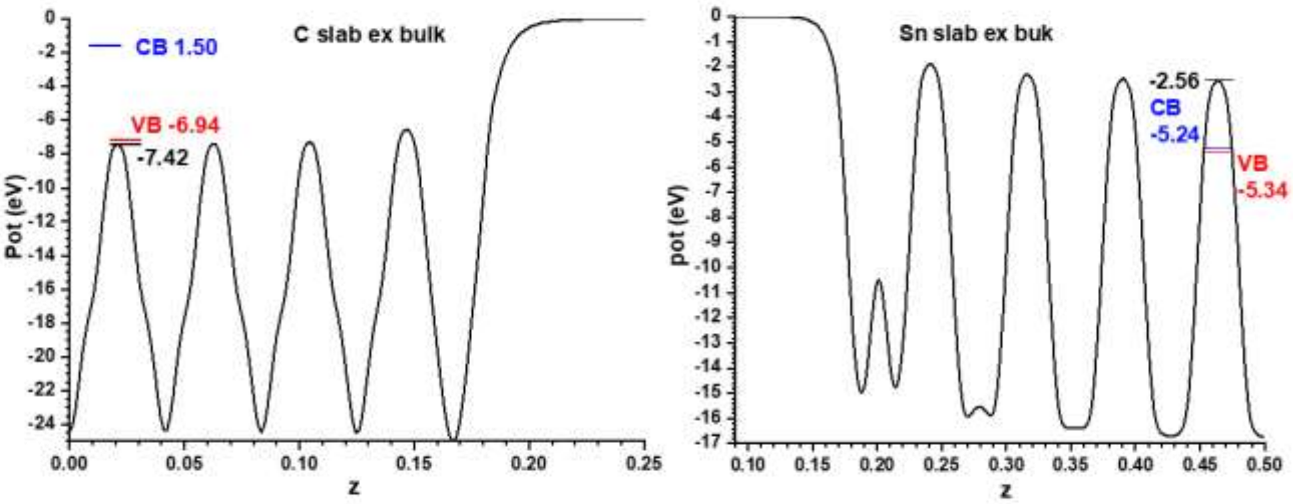

Figure S15 Structure of the diamond-type C and α-tin compound slab, including the Hartree potential profile and the positions relative to it of the VB and CB of both materials.

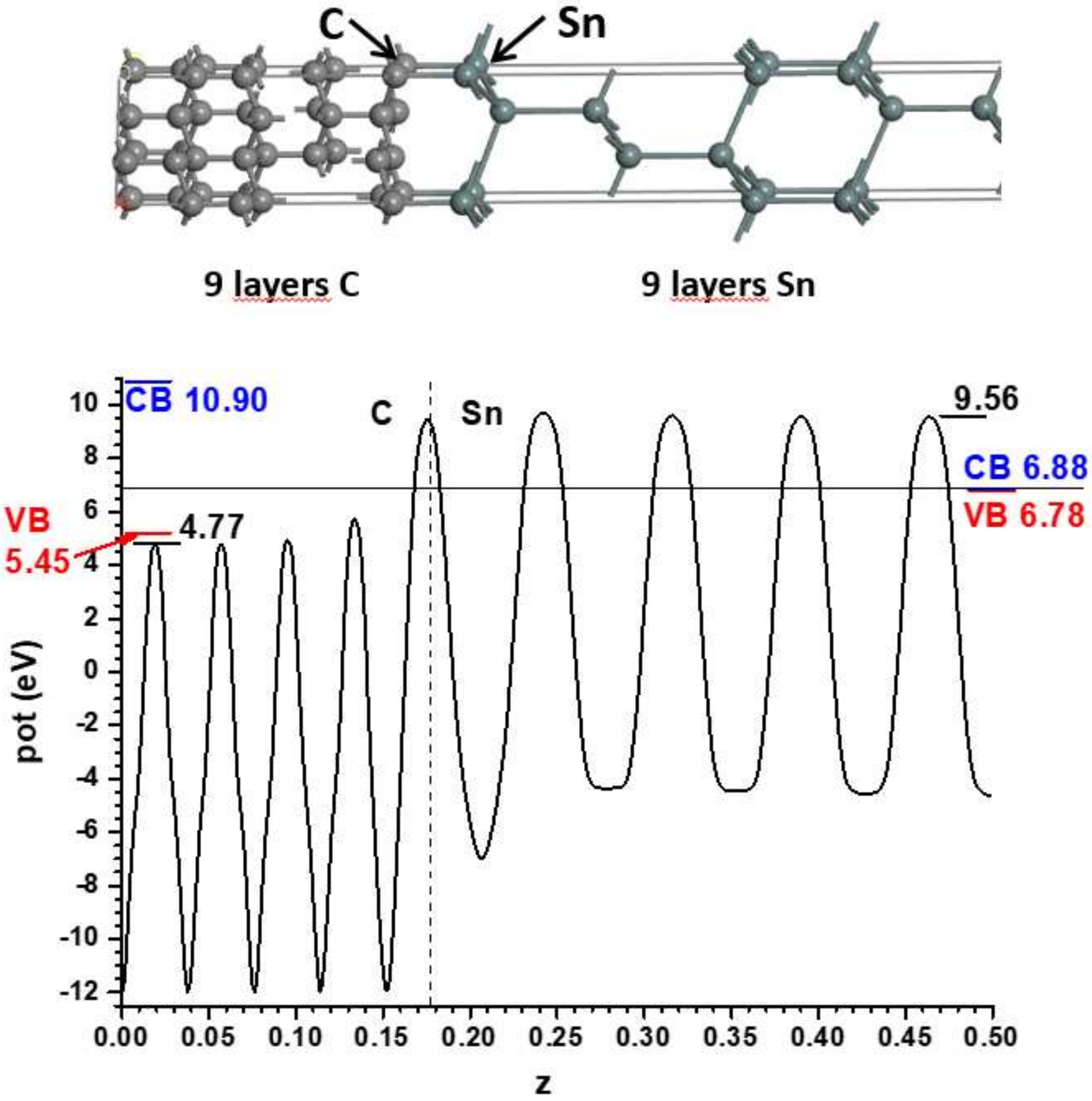

Figure S16 Charge density profile for the diamond-type C| α-tin compound slab, and its difference with the sum of the individual slab densities (keeping cell and atom positions unchanged).

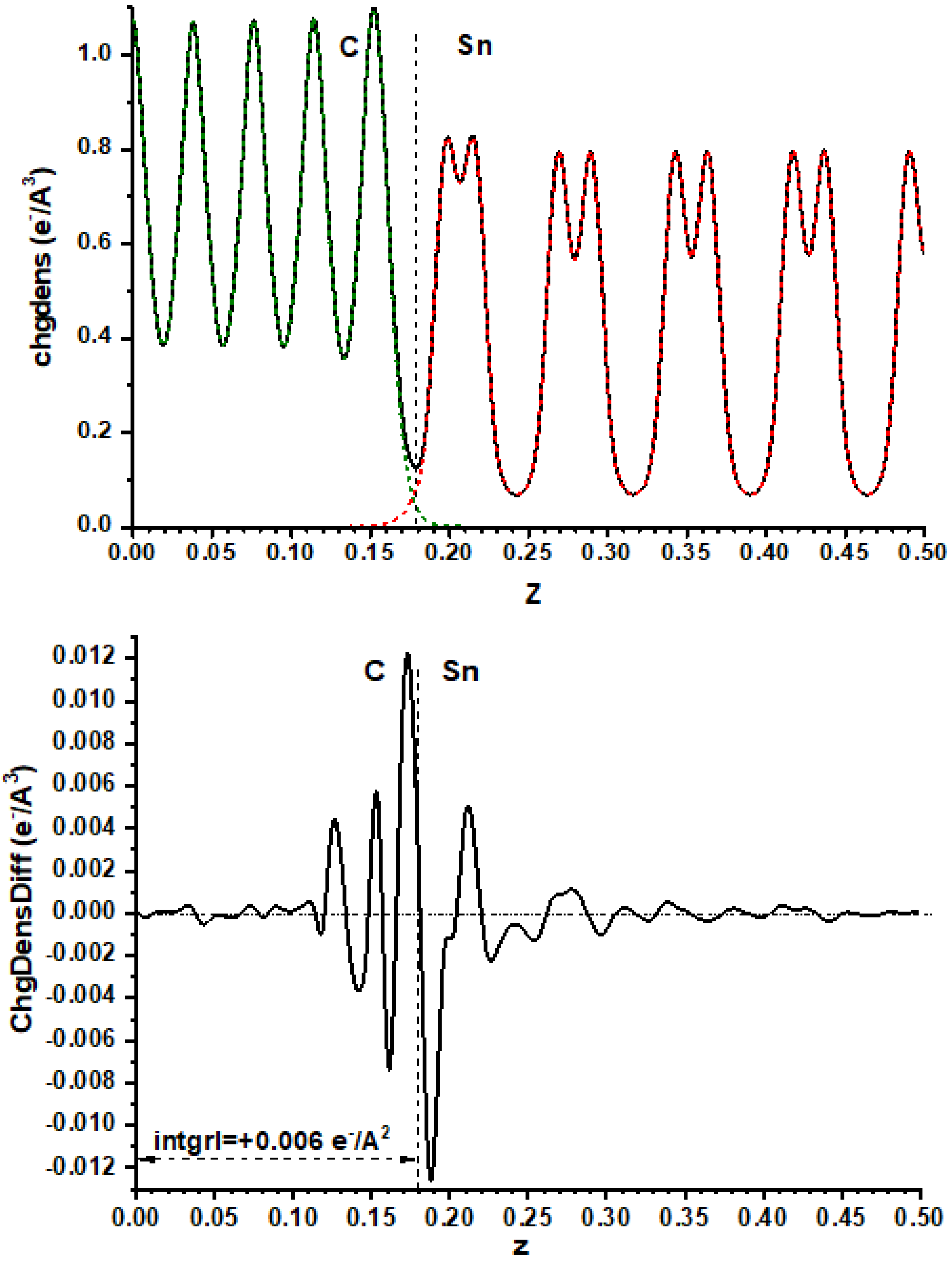

Supplement: Supplementary file 1 [file nanomaterials-11-01581-s001.zip › nanomaterials-1263218-supplementary.pdf]
